# Multi‐Metallic Nanosheets Reshaping Immunosuppressive Tumor Microenvironment through Augmenting cGAS‐STING Innate Activation and Adaptive Immune Responses for Cancer Immunotherapy

**DOI:** 10.1002/advs.202403347

**Published:** 2024-08-09

**Authors:** Yuxuan Peng, Shuang Liang, Dan Liu, Kongshuo Ma, Kaiqing Yun, Mengli Zhou, Linna Hai, Mengdi Xu, Yiyang Chen, Zhaohui Wang

**Affiliations:** ^1^ State Key Laboratory of Bioactive Substance and Function of Natural Medicines Institute of Materia Medica Chinese Academy of Medical Sciences & Peking Union Medical College Beijing 100050 China; ^2^ Beijing Key Laboratory of Drug Delivery Technology and Novel Formulation Institute of Materia Medica Chinese Academy of Medical Sciences & Peking Union Medical College Beijing 100050 China

**Keywords:** cancer immunotherapy, cGAS‐STING signaling pathway, immunosuppressive tumor microenvironment, layered double hydroxides

## Abstract

The highly immunosuppressive tumor microenvironment (TME) restricts the efficient activation of immune responses. To restore the surveillance of the immune system for robust activation, vast efforts are devoted to normalizing the TME. Here, a manganese‐doped layered double hydroxide (Mn‐LDH) is developed for potent anti‐tumor immunity by reversing TME. Mn‐LDH is synthesized via a one‐step hydrothermal method. In addition to the inherent proton neutralization capacity of LDH, the introduction of manganese oxide endows LDH with an additional ability to produce oxygen. Mn‐LDH effectively releases Mn^2+^ and Mg^2+^ upon exposure to TME with high levels of H^+^ and H_2_O_2_, which activates synthase‐stimulator of interferon genes pathway and maintains the cytotoxicity of CD8^+^ T cells respectively, achieving a cascade‐like role in innate and adaptive immunity. The locally administered Mn‐LDH facilitated a “hot” network consisting of mature dendritic cells, M1‐phenotype macrophages, as well as cytotoxic and helper T cells, significantly inhibiting the growth of primary and distal tumors. Moreover, the photothermal conversion capacity of Mn‐LDH sparks more robust therapeutic effects in large established tumor models with a single administration and irradiation. Overall, this study guides the rational design of TME‐modulating immunotherapeutics for robust immune activation, providing a clinical candidate for next‐generation cancer immunotherapy.

## Graphical abstract

Mn‐doped MgAl‐LDH is engineered to reprogram immunosuppressive tumor microenvironment through proton neutralization and oxygen generation. The released Mn^2+^ and Mg^2+^ initiate a robust cascade immune response, playing a dual role in innate and adaptive immunity by activating the cGAS‐STING signaling pathway and sustaining the cytotoxicity of CD8^+^ T cells, thus effectively inhibiting the growth of primary and distal tumors.

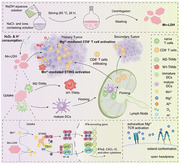

## 1 Introduction

As a revolutionary paradigm of cancer treatment, immunotherapy is known for its potential to inhibit tumor metastasis and recurrence.^[^ ^1^ ^]^ However, cancer immunotherapy still faces significant issues, including low response rates and immune resistance, due to the immune evasion triggered by the suppressive tumor microenvironment (TME).^[^ ^2^ ^]^ The TME is an intricated network surrounding tumor cells, composed of various cell types, stroma, blood vessels, cytokines, and extracellular matrix.^[^ ^3^ ^]^ This harsh environment that supports the proliferation and progression of tumor cells often leads to hypoxia, low pH, elevated reactive oxygen species, high interstitial pressure, and lack of nutrient metal ions, promoting the secretion of immunosuppressive cytokines and the recruitment and proliferation of immune regulatory cell populations.^[^ ^4^ ^]^ As an illustrative example, tumor‐associated macrophages (TAMs) are polarized into immunosuppressive M2 phenotypes, and T helper cells are transformed into regulatory T (Treg) cells in hypoxic and acidic environments.^[^ ^5^ ^]^

Hence, targeting specific pathways and components to normalize the abnormal ecological niche of TME is effective in enhancing anti‐tumor immune responses and restoring cancer surveillance by the immune system.^[^ ^4a,d^ ^]^ For instance, neutralizing protons in TME or reversing hypoxia can repolarize TAMs from the M2 phenotype to a proinflammatory M1 phenotype that characterized by effective phagocytosis and restore the function of CD8^+^ T cells.^[^ ^6^ ^]^ In addition, considering the lack of specific functional metal cations may impede the activation and maturation of antitumor immune cells, strategies based on supplementing functional metal cations (such as Mn^2+^, Zn^2+^, Mg^2+^, Ca^2+^, etc.) have sparked intensive attention.^[^ ^7^ ^]^ For instance, the costimulatory molecule LFA‐1 on the surface of CD8^+^ T cells needs to adopt Mg^2+^ to induce its active conformation, thereby enhancing and maintaining the cytotoxicity of CD8^+^ T cells.^[^ ^8^ ^]^ Layered double hydroxide (LDH), as a nanocarrier being extensively explored in the realm of biomedicine, has the potential to normalize TME as well as support effective immune activation due to its capability to neutralize protons and replenish specific metal ions.^[^ ^9^ ^]^

The activation of innate immunity is a prerequisite for the initiation of immune response.^[^ ^10^ ^]^ Increasing evidence suggests that the cyclic GMP‐AMP synthase (cGAS)‐stimulator of interferon genes (STING) signaling pathway is an essential component of innate immunity against microbial pathogens and malignant cells.^[^ ^11^ ^]^ Double‐stranded DNA (dsDNA) originating from dying cancer cells enter the cytoplasm of antigen‐presenting cells and activate the cGAS‐STING signaling pathway, leading to the production of type I interferons (IFN‐I) along with other immune‐stimulating factors that promote anti‐tumor responses mediated by CD8^+^ T cells and NK cells.^[^ ^11^, ^12^ ^]^ Furthermore, cGAS‐STING activation facilitates the maturation of dendritic cells (DCs) and repolarization of M2‐TAMs to M1 phenotype.^[^ ^13^ ^]^ As a crucial nutrient metal ion involved in multiple physiological processes, the function of Mn^2+^ in innate immunity has been generally elucidated. Mn^2+^ enhances the sensitivity of cGAS toward dsDNA, as well as boosts STING protein activity by augmenting cGAMP‐STING binding affinity. In addition, Mn^2+^ itself activates cGAS independently of dsDNA for the activation of immune cells.^[^ ^14^ ^]^ Specific manganese oxide nanoparticles (e.g., MnO_2_ and Mn_3_O_4_) can be converted into Mn^2+^ in TME for STING activation. Notably, they also efficiently generate O_2_ by consuming H_2_O_2_, allowing the alleviation of TME hypoxia.^[^ ^15^ ^]^

**Scheme 1:**
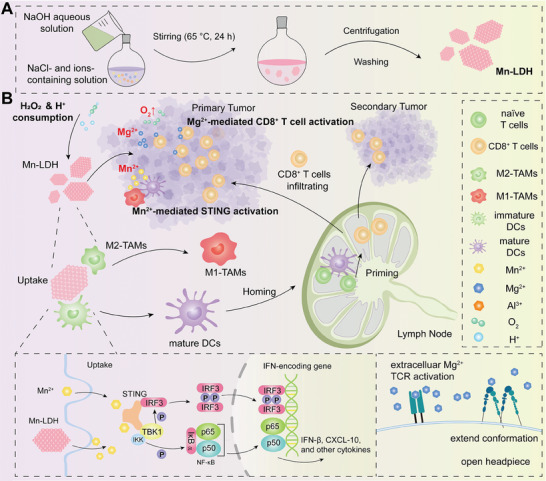
Schematic illustration of Mn‐LDH for cancer immunotherapy. A) Mn‐LDH composed of Mn, Mg, and Al was synthesized via a one‐step moderate hydrothermal method. B) Mn‐LDH modulated TME through consuming H^+^ and generating O_2_ and induced the activation of cGAS‐STING innate pathway and cascade adaptive immune response.

Herein, we reported the development of an engineered LDH nanoplatform to reshape the TME for efficient cancer immunotherapy by linking the multidimensional regulation of cascade immune activation. The LDH composed of Mn, Mg, and Al (Mn_3_O_4_‐doped LDH, referred to as Mn‐LDH hereafter) was fabricated via a simple moderate hydrothermal method (**Scheme** **1**A). The Mn‐LDH not only neutralized H^+^ but also consumed hydrogen peroxide (H_2_O_2_) to produce O_2_ for reprogramming the immunosuppressive TME. This reprogramming process that aimed at normalizing the TME helps to form a positive feedback loop^[^ ^16^ ^]^ with the subsequent release of metal ions, whereby Mn^2+^ and Mg^2+^ are capable of functioning as immune activators, further contributing to the amelioration of immunosuppressive TME. The Mn^2+^ and Mg^2^⁺ released in response to TME or intracellular matrix activated the cGAS‐STING signaling pathway and enhanced the activity of CD8^+^ T cells, respectively, thereby exerting cascading effects on innate and adaptive immunity. Therefore, this nanoplatform represents a cascaded TME‐normalizing reagent that facilitates to overcome immune evasion by activating both innate and adaptive immune responses. The Mn‐LDH led to potent tumor growth inhibition and prolonged survival time in breast and colon tumor models. Importantly, the photothermal conversion capability of Mn‐LDH was demonstrated and induced robust therapeutic effects in a large established tumor model with a single administration and irradiation. Together, the multifunctional nanosheets reversed the immunosuppressive microenvironment by promoting the activation of cGAS‐STING innate pathway and cascade adaptive immune response, providing a new platform to potentiate metalloimmunotherapy (Scheme 1B).

## 2 Results and Discussion

### 2.1 Preparation and Characterization of LDH and Mn‐LDH

**Figure 1:**
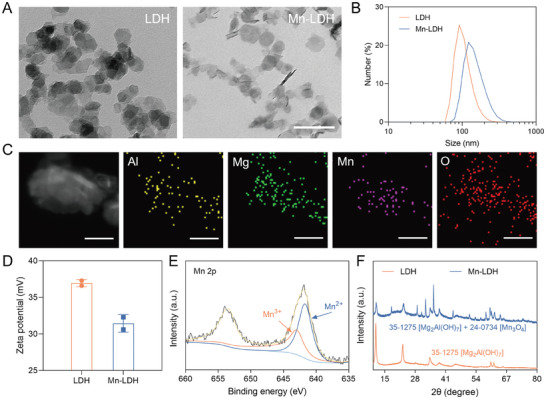
Characterization of LDH and Mn‐LDH. A) TEM images of LDH and Mn‐LDH. Scale bar: 200 nm. B) Size distribution of LDH and Mn‐LDH in deionized water (n = 3). C) Elemental mapping of Mn‐LDH. Scale bar: 50 nm. D) Zeta potential of LDH and Mn‐LDH (n = 2). E) XPS pattern of Mn 2p spectra of Mn‐LDH. F) XRD pattern of LDH and Mn‐LDH. All data are expressed as mean ± SD.

The LDH and Mn‐LDH were primarily synthesized using a one‐step hydrothermal method. As the optimal molar ratio of M^2+^/M^3+^ falls within the range of 2:1 to 4:1,^[^ ^17^ ^]^ we maintained a ratio of 3:1 to synthesize LDH (specifically, Mg:Al = 3:1). For Mn‐LDH, the molar ratio of Mn:Mg:Al was set to 1:2:1. The achieved molar ratios of synthesized LDH and Mn‐LDH were ≈2.5:1.0 and 1.2:2.3:1.0, respectively, which is consistent with the initial ratios (Table S1, Supporting Information). Transmission electron microscopy (TEM) revealed that Mn‐LDH exhibited a hexagonal‐like lamellar structure as LDH, with an average diameter of ≈50–100 nm (**Figure** **1**A). Dynamic light scattering (DLS) further demonstrated that Mn‐LDH had a hydrodynamic diameter of 184.2 ± 1.1 nm, slightly larger than LDH of 131.4 ± 0.2 nm (Figure 1B). Elemental mapping confirmed the presence of Mg, Al, Mn, and O elements in Mn‐LDH (Figure 1C). In contrast to the increase in particle size, the average potential of Mn‐LDH decreased from 37.0 ± 0.3 mV to 31.4 ± 0.9 mV (Figure 1D).

To determine the valence state of Mn and its doping form in Mn‐LDH, we analyzed the X‐ray photoelectron spectroscopy (XPS) and X‐ray diffraction (XRD) patterns of Mn‐LDH. As depicted in Figure S1 (Supporting Information), peaks corresponding to Mg 1s, O 1s, Al 2p, and Mn 2p are clearly observed. The binding energies at 641.3 eV and 653.0 eV are assigned to Mn 2p3/2 and Mn 2p1/2, respectively, corresponding to the Mn 2p3/2 peak of Mn_3_O_4_. The asymmetrical Mn 2p3/2 peak comprises two independent peaks at 640.8 eV and 642.1 eV, attributed to Mn^2+^ and Mn^3+^, respectively (Figure 1E). As shown by the XRD pattern in Figure 1F, the chemical structure of LDH corresponded to Mg_2_Al(OH)_7_, whereas Mn‐LDH was a hybrid of Mn_3_O_4_ and Mg_2_Al(OH)_7_. Collectively, we successfully constructed Mn‐LDH composed of Mn, Mg, and Al using a convenient one‐pot hydrothermal method, wherein Mn was doped into MgAl‐LDH in divalent and trivalent oxidation states.

### 2.2 TME‐Responsive Properties of Mn‐LDH In Vitro

**Figure 2:**
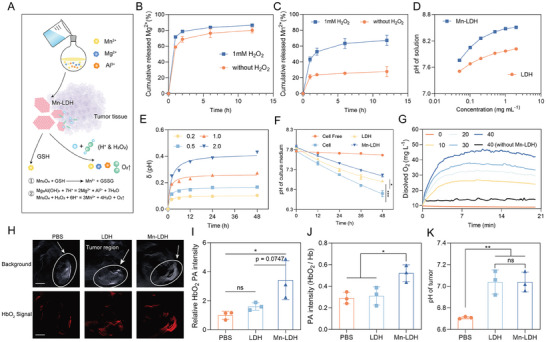
Mn‐LDH effectively responded to chemical stimuli in TME. A) Schematic illustration of the Mn‐LDH to release ions, neutralize protons, and produce oxygen in response to H^+^, H_2_O_2,_ and GSH. (B, C) Percentages of released Mg^2+^ B) and Mn^2+^ C) from Mn‐LDH over time in the PBS buffer (pH 6.5) with or without 1 mM H_2_O_2_, respectively (n = 3). D) pH of the deionized water containing different concentrations of LDH and Mn‐LDH. E) The change of pH in PBS buffer with different concentrations of Mn‐LDH at the indicated time. F) The pH of the 4T1 cell culture medium in the presence of LDH and Mn‐LDH (500 µg mL^−1^) at indicated time (n = 3). G) Oxygen generation from the decomposition of different concentrations of H_2_O_2_ (0, 10, 20, 30, 40 µm) after treatment with Mn‐LDH (40 µg mL^−1^). H) PA images of tumor tissues at 24 h after injection (Scale Bar: 10 mm). I,J) PA intensity of HbO_2_ (I) and HbO_2_/Hb ratio (J) at 24 h after injection (n = 3). K) pH value of tumor tissues in vivo 24 h after injection (n = 3). All data are expressed as mean ± SD, using GraphPad software for statistical analysis, and the differences were assessed using one‐way ANOVA with Tukey's post‐test. *P < 0.05, **P < 0.01, ***P < 0.001.

Considering the presence of elevated levels of protons (pH 6.5 to 6.8) and H_2_O_2_ (≈100 µm) in TME (**Figure** **2**A), we first investigated the release profile of Mn^2+^ and Mg^2+^ in PBS buffers with or without H_2_O_2_ at pH 6.5.^[^ ^18^ ^]^ As illustrated in Figure 2B, the cumulative release of Mg^2+^ witnessed a slight increase from 80.1% to 86.6% in the presence of H_2_O_2_ after 12 h. However, the cumulative release rate of Mn^2+^ in PBS was only 27.8% at 12 h, while 67.0% release was achieved in the presence of H_2_O_2_ (Figure 2C). This suggests that Mg^2+^ in Mn‐LDH is predominantly released through an acid‐sensitive mechanism, and the additional presence of H_2_O_2_ has a mild effect on the dissolution of the Mn‐LDH skeleton and thus Mg^2+^ release. In contrast, Mn^2+^ in Mn‐LDH exhibited superior responsiveness to H^+^ plus H_2_O_2_ than H^+^ alone, which can be attributed to the following chemical equation: Mn_3_O_4_ + H_2_O_2_ + 6H^+^ = 3Mn^2+^ + 4H_2_O + O_2_↑^[^ ^19^ ^]^

Due to the redox sensitivity of Mn_3_O_4_, Mn‐LDH also demonstrated responsiveness to glutathione (GSH), evident by the gradual fading of the brownish‐yellow color of Mn_3_O_4_ (Figure S2, Supporting Information).^[^ ^20^ ^]^ The aqueous solutions of LDH and Mn‐LDH exhibited alkaline characteristics (Figure 2D), as indicated by pH values of 7.8 and 8.5 for 50 and 1000 µg mL^−1^ of Mn‐LDH solutions, respectively, which were higher than those of LDH at the same concentrations (with pH values of 7.5 and 8.0, respectively). This phenomenon is possibly attributed to the additional proton absorption capacity of Mn_3_O_4_. Based on this, we next investigated whether Mn‐LDH possessed an intrinsic capacity to neutralize acidic buffers and delay the acidification of the cell culture medium. The results demonstrated that Mn‐LDH (500 µg mL^−1^) increased the pH value of PBS buffer (6.5) by 0.2 and elevated the pH value of 4T1 cell medium from 6.7 to 7.2 within 48 h (Figure 2E,F).

Previous studies indicated that Mn_3_O_4_ can generate oxygen in the presence of H^+^ and H_2_O_2_,^[^ ^15^, ^21^ ^]^ we next evaluated the oxygen generation capacity of Mn‐LDH. As depicted in Figure 2G and Figure S3 (Supporting Information), Mn‐LDH exhibited a robust capability to generate oxygen. When the Mn‐LDH concentration was held constant at 40 µg mL^−1^, and the H_2_O_2_ concentration was gradually increased from 0 to 40 µm, the maximum dissolved O_2_ concentration measured in water was 9.7, 26.9, 33.3, 37.7, and 46.5 mg L^−1^, respectively. These results indicated a positive correlation between O_2_ generation induced by Mn‐LDH and H_2_O_2_ concentration. Similarly, a concentration‐dependent O_2_ content was observed across various concentrations of Mn‐LDH (Figure S4, Supporting Information). Moreover, at a concentration of 50 µg mL^−1^ for Mn‐LDH and 40 µm for H_2_O_2_, the maximum dissolved oxygen concentration in water for Mn‐LDH and LDH was 54.8 and 17.0, respectively. This suggested that the robust oxygen production capability of Mn‐LDH arose from the incorporation of Mn_3_O_4_, while LDH generated a minimal level of O_2_.

To further elucidate the TME‐response and ‐regulating capacity of Mn‐LDH in vivo, we investigated whether Mn‐LDH alleviates the hypoxic and acidic TME in 4T1 tumor‐bearing mice.^[^ ^22^ ^]^ Photoacoustic imaging (PA) results revealed that Mn‐LDH significantly augmented the PA signal of oxygenated hemoglobin (HbO_2_), while LDH‐treated mice exhibited a comparable PA signal of HbO_2_ to PBS‐treated mice (Figure 2H,I), suggesting the introduction of Mn_3_O_4_, not LDH own, improved the local oxygen supply. In addition, the enhanced oxygenated/deoxygenated hemoglobin (HbO_2_/Hb) ratio further indicated that Mn‐LDH efficiently relieved tumor hypoxia (Figure 2J). To investigate whether LDH and Mn‐LDH could alleviate the acidic TME through acid‐base neutralization, we measured the pH value of the tumor after administration utilizing a pH microelectrode. As shown in Figure 2K, the pH of the tumor increased from 6.71 to 7.04 after being treated with LDH or Mn‐LDH. This data demonstrated the effective neutralizing capacity of LDH and Mn‐LDH, which was in line with the in vitro experiments.

Taken together, Mn‐LDH demonstrated a robust response to the TME and facilitated the programmable release of functional ions (Mn^2+^ and Mg^2+^), allowing to mitigate the immunosuppressive microenvironment by alleviating the acidic pH and hypoxia.

### 2.3 Mn‐LDH‐Induced BMDC Maturation and cGAS‐STING Signaling Pathway Activation

**Figure 3:**
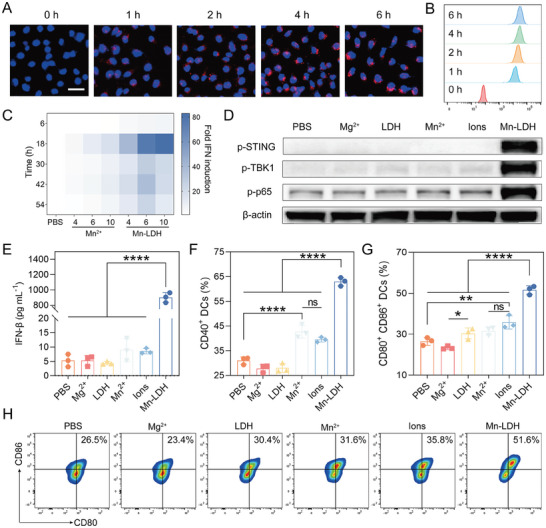
Mn‐LDH induced BMDC maturation and cGAS‐STING signaling pathway activation. A) Fluorescence microscope images of DC2.4 cells treated by Mn‐LDH. Red: BSA‐Cy5‐labeled Mn‐LDH. Blue: nuclei (scale bar: 40 µm). B) Fluorescence‐activated cell sorting (FACS) histograms of Mn‐LDH that internalized into DC2.4 cells. C) Mn‐LDH triggered the activation of the cGAS‐STING signaling pathway in RAW‐Lucia ISG cells and induced the secretion of IFN‐I (4, 6, 10 represent that the concentration of manganese, µg mL^−1^, n = 4). D) Western blot analysis of STING activation in BMDCs with different treatments. E) Secretion of IFN‐β after BMDCs cocultured with different formulations (n = 3). F,G) The expression of costimulatory molecules CD40 (F), CD80, and CD86 (G) on BMDCs (n = 3). H) Representative FACS plots of CD80^+^ CD86^+^ BMDCs. All data are expressed as mean ± SD, using GraphPad software for statistical analysis, and the differences were assessed using one‐way ANOVA with Tukey's post‐test. *P < 0.05, **P < 0.01, ****P < 0.0001.

We subsequently investigated the cellular uptake and immune activation of Mn‐LDH. Given the high efficiency of LDH in adsorbing biological macromolecules, we labeled Mn‐LDH using Cy5‐labeled bovine serum albumin (referred to as BSA‐Cy5 hereafter). As anticipated, Mn‐LDH exhibited efficient adsorption of BSA‐Cy5, achieving a loading capacity of over 50%, while maintaining colloidal stability. This observation also suggested the potential of Mn‐LDH for the efficient delivery of biomacromolecules. Fluorescence microscopy and flow cytometry results revealed a robust red fluorescence signal following 1 h incubation of BSA‐Cy5 labeled Mn‐LDH with DC2.4 cells (**Figure** **3**A,B; Figures S5 and S6, Supporting Information). The intracellular fluorescence intensity exhibited a gradual increase when extending the incubation time to 6 h, indicating a sustained internalization of Mn‐LDH.

Activation of the STING signaling pathway induces the secretion of IFN‐I and other inflammatory cytokines, thereby triggering adaptive responses to enhance cancer immunotherapy.^[^ ^23^ ^]^ To demonstrate the activation of the cGAS‐STING pathway first, we employed RAW‐Lucia ISG cells as reporter cells to observe IFN‐I production. The heat map in Figure 3C illustrated that Mn‐LDH induced a more robust IFN‐I response compared to free Mn^2+^ at each corresponding concentration. The reporter cells produced an 83.4‐fold increase in IFN‐I in Mn‐LDH (containing 10 µg mL^−1^ Mn^2+^)‐treated cells compared to PBS‐treated cells after 18 h of co‐incubation, whereas the 18.9‐fold increment over PBS was shown in free Mn^2+^‐treated cells. In Figures S7 and S8 (Supporting Information), it was observed that the Ions group had a less favorable effect on the generation of IFN‐I compared to the Mn^2+^ alone, exhibiting a 4.6‐fold enhancement over the PBS‐treated group at 24 h. This enhancement was considerably lower than that of the Mn^2+^‐treated group (35.9‐fold) and the Mn‐LDH‐treated group (87.1‐fold).

In addition, the downstream signaling of the STING pathway was assessed through Western Blot analysis. Activated STING upregulates TANK‐binding kinase 1 (TBK1), facilitating TBK1 autophosphorylation, recruitment of interferon regulatory factor 3 (IRF3), and STING phosphorylation.^[^ ^24^ ^]^ The TBK1‐mediated phosphorylation of IRF3 ultimately enhances the production of type I interferons and various other inflammatory mediators.^[^ ^24^ ^]^ Furthermore, activated STING also promotes NF‐κB (p50 and p65) phosphorylation and nuclear translocation, collaborating with p‐IRF3 to induce the expression of inflammatory mediators such as IFNs, tumor necrosis factors, interleukin (IL)−1β, and IL‐6.^[^ ^25^ ^]^ Results demonstrated that Mn‐LDH significantly augmented the phosphorylation levels of STING, TBK‐1, and NF‐κB p65, indicating that Mn‐LDH activated the cGAS‐STING signaling pathway via both the TBK1 and NF‐κB pathways (Figure 3D). Additionally, the expression of phosphorylated NF‐κB p65 was slightly increased in the Mn^2+^ and Ions groups (the mixtures of Mn^2+^ and Mg^2+^ corresponding to the ion ratio of Mn‐LDH, hereafter “Ions”), both being 1.3‐fold over that of PBS, respectively. Consequently, we quantified IFN‐β secretion using enzyme‐linked immunosorbent assay (ELISA) to confirm the activation of cGAS‐STING signaling pathway in bone marrow‐derived dendritic cells (BMDCs). Figure 3E showed 900.6 pg mL^−1^ of IFN‐β was observed in the Mn‐LDH‐stimulated medium supernatant, reaching 169.9‐fold over that of the control group. In comparison, only negligible increases in IFN‐β were observed in the Mn^2+^‐ and Ions‐stimulated supernatants.

The activation of STING pathway facilitates the maturation and activation of DCs, which play a crucial role in initiating innate immunity and triggering subsequent immune responses.^[^ ^26^ ^]^ To this end, we cultivated BMDCs and subjected them to various treatments. Specifically, Mg^2+^ and LDH exhibited minimal up‐regulation of CD40 expression on BMDCs compared to the PBS group, with CD40^+^ BMDC ratios of 27.8% and 28.0%, respectively (Figure 3F; Figure S9, Supporting Information). In contrast, cells treated by Mn^2+^, Ions, and Mn‐LDH displayed an increased proportion of CD40^+^ BMDCs to 42.7%, 39.5%, and 63.0%, respectively (Figure S10, Supporting Information). Similar results were also validated in the proportions of CD80^+^ CD86^+^ BMDCs. As shown in Figure 3G,H, the treatment by PBS, Mg^2+^, LDH, Mn^2+^, Ions, and Mn‐LDH resulted in the proportion of CD80^+^ CD86^+^ BMDCs reaching 26.5%, 23.4%, 30.4%, 31.6%, 35.8%, and 51.6%, respectively. LDH modestly elevated the proportion of CD80^+^ CD86^+^ BMDCs, probably attributed to its widely reported adjuvant‐like activity.^[^ ^9^, ^27^ ^]^ Expectedly, the maturation of BMDC was dependent on the concentration of Mn‐LDH (Figure S11, Supporting Information). Taken together, Mn‐LDH exhibited robust adjuvant‐like activity through STING activation, promoting the initiation of innate immunity and the secretion of inflammatory cytokines to achieve potent cancer immunotherapy.

### 2.4 In Vivo Therapeutical Efficacy of Mn‐LDH and Mechanism Analysis

Before validating the in vivo therapeutic efficacy, we first tested the safety of Mn‐LDH both in vitro and in vivo. As shown in Figure S12 (Supporting Information), Mn‐LDH demonstrated a hemolysis rate of less than 1% in a concentration range of 0.1–8.0 mg mL^−1^, indicating the biocompatibility of Mn‐LDH. In addition, no obvious weight loss was occurred during the treatment in healthy mice (Figure S13, Supporting Information). Moreover, there was negligible change in hepatic and renal function of Mn‐LDH‐treated mice compared with PBS‐treated mice (Figure S14, Supporting Information), revealing the Mn‐LDH's excellent biosafety profile preliminarily.

**Figure 4:**
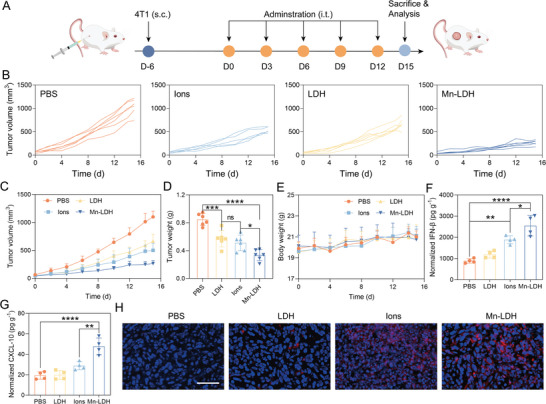
In vivo therapeutical efficacy of LDH, Ions, and Mn‐LDH in the 4T1 TNBC tumor model. A) Schematic illustration of the intratumoral injection of PBS, LDH, Ions, and Mn‐LDH. B) The individual tumor volume of mice with different treatments at the indicated time (n = 6). D) The average tumor volume of mice at the indicated time was treated with different formulations (n = 6). C) The body weights of 4T1‐bearing mice at the indicated time. E) The tumor weight of excised 4T1 tumors. (F, G) Quantitative analysis of the secretion of IFN‐β F) and CXCL‐10 G) in 4T1 tumors (n = 4). H) Immune fluorescence staining images of recruited p‐STING^+^ cells in the frozen tumor section. Blue: nuclei. Red: p‐STING (scale bar: 100 µm). All data are expressed as mean ± SD, using GraphPad software for statistical analysis, and the differences were assessed using one‐way ANOVA with Tukey's post‐test. *P < 0.05, **P < 0.01, ***P < 0.001, ****P < 0.0001.

Encouraged by the superior capability of Mn‐LDH in the reshaping of TME and immune activation, we endeavored to investigate the anti‐tumor efficacy in vivo. For this, 4T1 triple‐negative breast cancer (TNBC) model was utilized, which is known for poor immunogenicity, invasiveness, and propensity for spontaneous metastasis to distal organs.^[^ ^28^ ^]^ As illustrated in **Figure** **4**A, PBS, LDH, Ions, and Mn‐LDH were administrated intratumorally (i.t.) every 3 days. Following 15 days from the first i.t. administration, the tumor volumes for PBS, LDH, Ions, and Mn‐LDH treated mice were recorded as 1100.4 mm^3^, 668.2 mm^3^, 501.8 mm^3^, and 265.4 mm^3^, respectively (Figure 4B,C). Intriguingly, LDH and Ions exhibited mild inhibition on tumor progression, with tumor growth inhibition at 32.5% and 39.6%, respectively. Mn‐LDH achieved a significantly higher inhibition rate (60.4%) than PBS (P < 0.0001), LDH (P = 0.0029), and Ions (P = 0.0290) (Figure 4D; Figure S15, Supporting Information). The superior tumor growth inhibition observed with Mn‐LDH compared to LDH could be attributed to the essential role of introduced Mn in initiating innate immunity. Meanwhile, Mn‐LDH inhibited tumor growth more efficiently than Ions, likely due to the following factors: i) Mn‐LDH exhibited enhanced internalization by immune cells; ii) Mn‐LDH demonstrated efficacy in mitigating the suppressive TME, as validated through in vitro experiments. In addition, the body weight of mice across different groups displayed no discernible differences, indicating the safety of LDH, Ions, and Mn‐LDH at the predetermined dose and frequency of administration (Figure 4E). The biocompatibility of Mn‐LDH was further supported by Hematoxylin and Eosin (H&E) staining of vital organs, including the heart, liver, spleen, lung, and kidney (Figure S16, Supporting Information).

To investigate the correlation between the anti‐tumor effect induced by Mn‐LDH and STING activation, we examined STING‐related cytokines via ELISA. As shown in Figure 4F, the IFN‐β in the tumor supernatant of Mn‐LDH‐treated mice yielded a concentration of 2547.3 pg g^−1^ (i.e., IFN‐β concentration per gram of tumor tissue), representing a 2.9‐fold increase compared to PBS‐treated mice. Similarly, Ions‐treated mice exhibited a concentration of 1883.8 pg g^−1^, which was 2.1 times higher than the PBS group. A similar trend was observed in the concentration of CXCL‐10 (Figure 4G), where the tumor supernatant from Mn‐LDH‐treated mice reached 47.8 pg g^−1^, which was 2.5, 2.3, and 1.6 times higher than those treated with PBS, LDH, and Ions, respectively. As anticipated, the LDH group did not show significant up‐regulation of IFN‐β and CXCL‐10, which was in line with the STING protein phosphorylation and previous in vitro studies. Through immunofluorescence staining of frozen tumor tissue sections, mice treated with Mn‐LDH displayed robust red fluorescence of p‐STING, while faint fluorescence was observed in the Ions group (Figure 4H).

**Figure 5:**
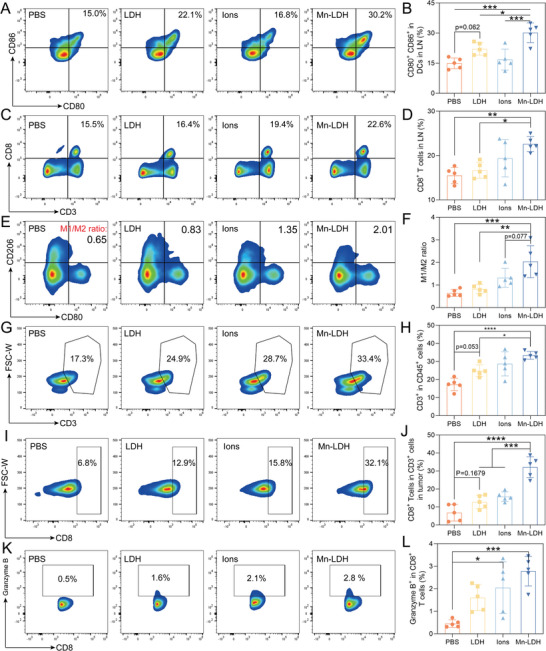
Mn‐LDH prominently boosted anti‐tumor immune responses by innate and adaptive immunity activation. A,B) Representative FACS plots (A) and quantitative analysis B) of CD80^+^ CD86^+^ DCs in tumors. C,D) Representative FACS plots (C) and quantitative analysis D) of CD8^+^ T cells in LNs. E,F) Representative FACS plots E) and quantitative analysis F) of the ratio of M1‐TAMs (CD80^+^) and M2‐TAMs (CD206^+^) in tumors. G,H) Representative FACS plots (G) and quantitative analysis (H) of the proportions of CD3^+^ T cells in CD45^+^ cells in tumors. I,J) Representative FACS plots (I) and quantitative analysis (J) of the proportions of CD8^+^ T cells in CD3^+^ cells in tumors. K,L) Representative FACS plots (K) and quantitative analysis (L) of the proportions of Granzyme B^+^ in CD8^+^ T cells in tumors. All data are expressed as mean ± SD (n = 5), using GraphPad software for statistical analysis, and the differences were assessed using one‐way ANOVA with Tukey's post‐test. *P < 0.05, **P < 0.01, ***P < 0.001, ****P < 0.0001.

Building upon the superior anti‐cancer efficacy, our subsequent investigation was aimed to elucidate the detailed mechanism. To investigate the consistency of DC maturation between the in vivo and in vitro effects, lymph nodes (LNs) were isolated, ground into single‐cell suspensions, and subjected to flow cytometric analysis. The Mn‐LDH significantly enhanced the ratio of CD80^+^ CD86^+^ DCs (30.2%), which is 2.1‐fold, 1.4‐fold, and 1.6‐fold over the PBS, LDH, and Ions‐treated groups, respectively (**Figure** **5**A,B). Consistent with in vitro findings, LDH and Ions also elicited modest increases in DC maturation with ratios at 22.1% and 16.8%, respectively. Furthermore, we observed a significant augmentation in the proportion of CD8^+^ T cells in the LNs of Mn‐LDH‐treated mice, rising from 15.5% to 22.6% compared to the PBS group (Figure 5C,D; Figure S17, Supporting Information).

To assess the potential of LDH, Ions, and Mn‐LDH in modulating the immunosuppressive TME, we analyzed the phenotype of TAMs using flow cytometry. Typically, tumors were dissociated with collagenase I and deoxyribonuclease I to obtain a single‐cell suspension. M1 macrophages expressing CD80 are associated with promoting tumor phagocytosis, while M2 macrophages expressing CD206 are linked to tumor growth (Figure S18, Supporting Information). As depicted in Figure 5E,F and Figure S19 (Supporting Information), Mn‐LDH significantly down‐regulated the expression of CD206. The M1/M2 ratio in mice treated with PBS, LDH, Ions, and Mn‐LDH was 0.6, 0.8, 1.3, and 2.0, respectively. As anticipated, the proton neutralization capacity of LDH modestly increased the M1/M2 ratio. Ions, with a 2.1‐fold increase in the M1/M2 ratio, suggested that the activation of the STING pathway could repolarize M2‐TAMs. Moreover, the ratio of CD8^+^ T cells to Tregs was prominently increased 12.0 times after being treated with Mn‐LDH over PBS (Figure S20, Supporting Information). The potent effect of Mn‐LDH indicated its versatility in STING activation, oxygen production, and acid neutralization, which synergistically contributed to the reversal of suppressive TME.

As shown in Figure 5G,H, and Figure S21 (Supporting Information), LDH, Ions, and Mn‐LDH increased the ratio of matured T cells (CD3^+^ cells) in CD45^+^ cells at 1.4‐, 1.6‐, and 1.9‐fold over PBS group, suggesting the enhanced adaptive immunity. The immunofluorescence of CD3 expression in tumor tissues was consistent with that of FACS data. (Figure S22, Supporting Information). Furthermore, Mn‐LDH treatment resulted in a significant increase in the infiltration of CD4^+^ and CD8^+^ T cells in the TME, crucial components of the adaptive immune response (Figure 5I,J; Figure S23A, Supporting Information). LDH‐treated mice also demonstrated a 1.8‐fold increase in CD4^+^ T cell infiltration and a 1.8‐fold increase in CD8^+^ T cell infiltration compared to PBS‐treated mice. However, 2.5‐fold and 4.7‐fold infiltration of CD4^+^ and CD8^+^ T cells was observed in Mn‐LDH‐treated mice. This suggested that the cascade immune activation induced by the additional introduction of Mn could trigger a more robust adaptive immune response. Additionally, the number of IFN‐γ^+^ CD8^+^ T cells increased significantly in Mn‐LDH‐treated mice, which was 13.0 times over that in PBS‐treated mice (Figure S23B, Supporting Information). Additionally, following Mn‐LDH treatment, the proportion of Granzyme B^+^ CD8 T cells increased by 5.6‐fold, rising from 0.5% to 2.8% (Figure 5K,L). Comparable increases in the infiltration of Granzyme B^+^ CD8 T cells were also noted in LDH‐ and Ions‐treated mice. Considering that CD8^+^ T cells constitute a major source of pro‐inflammatory factors such as IFN‐γ and Granzyme B,^[^ ^29^ ^]^ our findings suggested that Mn‐LDH effectively converted a “cold” 4T1 tumor into a “hot” one. These data demonstrate the significance of Mn‐LDH in bridging the innate and adaptive immune response.

In summary, Mn‐LDH facilitated the sequential activation of both innate and adaptive immunity. The integration of tumor microenvironment‐modulating capacity with the potent adjuvant‐like activity of Mn resulted in a substantial enhancement of anti‐tumor effects.

### 2.5 Mn‐LDH Effectively Inhibited both Primary Tumors and Distal Tumors in the Colorectal Cancer CT26 Model

**Figure 6:**
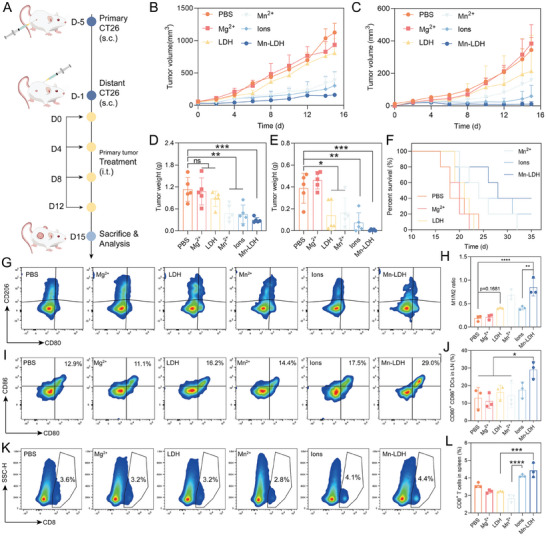
Mn‐LDH exhibited remarkable inhibition on both primary tumors and distal tumors in the colorectal cancer CT26 model. A) Schematic illustration of the i.t. injection of different formulations in CT26‐bearing mice. B,C) The average volume of the primary tumor (B) and distal tumor (C) of mice at the indicated time (n = 5). D,E) The tumor weight of excised primary (D) and distal (E) tumors on day 20 after primary tumor implantation (n = 5). F) The survival rate of mice with various treatments. G,H) Representative FACS plots G) and quantitative analysis H) of the ratio of M1‐TAMs (CD80^+^) and M2‐TAMs (CD206^+^) in tumor (n = 3). I,J) Representative FACS plots (I) and quantitative analysis (J) of CD80^+^ CD86^+^ DCs in LNs (n = 3). (K, L) Representative FACS plots K) and quantitative analysis L) of CD8^+^ T cells in spleens (n = 3). All data are expressed as mean ± SD, using GraphPad software for statistical analysis, and the differences were assessed using one‐way ANOVA with Tukey's post‐test. *P < 0.05, **P < 0.01, ***P < 0.001, ****P < 0.0001.

Subsequently, we further evaluated the therapeutic effect of Mn‐LDH on both primary and distal tumors in a colorectal cancer CT26 model. To investigate whether Mg^2+^ and Mn^2+^ could synergistically amplify immunotherapy in a cascade manner, we introduced free Mg^2+^ and free Mn^2+^ groups based on the aforementioned 4T1 TNBC model. As depicted in **Figure** **6**A, CT26 colorectal mice with tumors on both flanks received four doses of Mn‐LDH within the primary (right side) tumor. After 20 days of tumor implantation, Mn‐LDH exhibited a remarkable tumor growth inhibition ratio at 74.1% and 98.3% for primary and distal tumors, respectively (Figure 6B–E; Figure S24, Supporting Information), which was superior to free Mg^2+^ (almost negligible inhibition), LDH (22.9% and 64.0% inhibition), free Mn^2+^ (56.1% and 58.2% inhibition), and Ions (59.9% and 81.3% inhibition). Notably, Mg^2+^ exhibited minimal inhibition of both primary and distal tumor progression, while LDH effectively inhibited the growth of primary tumors, particularly distal tumors. This observation may be attributed to the higher accumulation of LDH in tumor tissue and increased internalization. Interestingly, we observed that the primary tumor weight in mice treated with Ions was not significantly lower than that in mice treated with free Mn^2+^ (456.0 mg and 498.0 mg, respectively). However, the weight of distal tumors in mice treated with Ions was only 44.7% of that in the free Mn^2+^ group. Moreover, Mn‐LDH significantly extended the survival time of mice (Figure 6F). In line with the result from 4T1 tumor model, Mn‐LDH did not induce any weight loss (Figure S25, Supporting Information).

Subsequently, we conducted an in‐depth investigation into the mechanism underlying tumor inhibition and the abscopal effects of Mn‐LDH. The M1/M2 ratio in tumors of Mn‐LDH‐treated mice was 0.9, with ratios of 0.2, 0.2, 0.4, 0.7, and 0.4 in PBS, Mg^2+^, LDH, Mn^2+^, and Ions treated mice, respectively (Figure 6G,H). The M1/M2 ratio in Mg^2+^‐treated mice indicated its failure to upregulate the ratio, while Mn^2+^‐mediated STING activation predominantly drove effective repolarization. A comparison of the M1/M2 ratio in Mg^2+^ and LDH‐treated mice suggested that proton neutralization also facilitated repolarization. Furthermore, we simultaneously assessed the maturation of DCs within the tumor. As anticipated, the proportion of CD80^+^ DCs (labeled with CD11c, distinct from F4/80‐labeled TAMs) increased from 1.2% to 6.0% (Figure S26A). In the LNs, Mn‐LDH treatment resulted in proportions of CD80^+^ CD86^+^ DCs at 29.0%, which were 2.2 times higher than the PBS‐treated group (Figure 6I,J). Additionally, the trends in DC maturation observed in LDH, Mn^2+^, and Ions‐treated mice aligned with those in in vitro experiments and the 4T1 TNBC models.

To evaluate the activation of the adaptive immune system and elucidate the mechanism of the abscopal effect, we quantified the infiltration of CD8^+^ cytotoxic T cells in the LNs and spleens. Mn‐LDH significantly upregulated CD8^+^ T cells in LNs and spleen by 1.8‐fold and 1.5‐fold over PBS, respectively, validating the rationale behind the abscopal effect of Mn‐LDH. Building upon these findings, we attempted to elucidate the abscopal effects between Mn^2+^ and Ions. After Mn^2+^ treatment, the infiltration of CD8^+^ T cells in the spleen of mice resembled that of the PBS‐treated group (Figure 6K,L; Figure S27, Supporting Information). Conversely, mice treated with Ions demonstrated an increased infiltration of CD8^+^ T cells in the spleen from 2.8% to 4.1%. Additionally, a slight upregulation of the ratio of CD8^+^ T cells in Ions to Mn^2+^ was observed in lymph nodes (Figure S26B, Supporting Information). Moreover, the proportion of NK cells (CD3^−^ CD49b^+^) in LNs increased from 5.1% to 8.1%, as depicted in Figure S26C (Supporting Information). Integrating these results, we preliminarily conclude that Mg^2+^ plays a pivotal role in systemic adaptive immunity subsequently to Mn^2+^‐induced innate immunity, as evidenced by the disparities in abscopal effects. Furthermore, through the examination of HIF‐1α expression in primary tumor tissues treated with PBS, LDH, and Mn‐LDH, we observed that Mn‐LDH effectively alleviates hypoxia (Figure S28, Supporting Information). In summary, both LDH and free Mn^2+^ demonstrated tumor growth inhibition, which was further amplified by the Mn^2+^ and Mg^2+^ mediate cascade immune response of Mn‐LDH. Importantly, the additional capacity for TME regulation endows Mn‐LDH with superior immune reprogramming compared to Ions, validating our initial hypothesis.

### 2.6 Photothermal Conversion Capacity and Photo‐Enhanced Immunotherapeutic Effects of Mn‐LDH in Established Tumors

**Figure 7:**
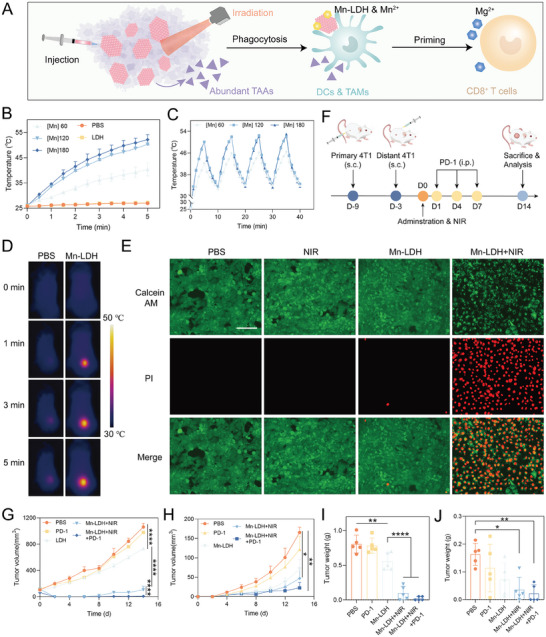
Photothermal conversion capacity and photo‐enhanced immunotherapeutic effects of Mn‐LDH. A) Scheme illustrates that of photo‐enhanced immunotherapy abundant TAAs produced after mild irradiation enhanced the Mn‐LDH‐mediated metalloimmunotherapy. B) Temperature change when different concentrations of Mn‐LDH were irradiated by laser with 1.5 W cm^−2^ at the indicated time (n = 3). C) Photothermal stability of Mn‐LDH for successive 4 cycles of on/off laser irradiation. D) Infrared thermal images of mice during laser irradiation for 5 min (1 W cm^−2^). E) Live/dead cell assay of Mn‐LDH. Green: Calcein AM. Red: PI. 808 nm laser was applied for 10 min in 1.5 W cm^−2^. F) Schematic illustration of the intratumoral injection of different formulations in 4T1‐bearing mice. G,H) The average volume of primary tumor (G) and distal tumor (H) of mice with different treatments (n = 5). I,J) The tumor weight of excised primary (H) and distal (I) tumors (n = 5). All data are expressed as mean ± SD, using GraphPad software for statistical analysis, and the differences were assessed using one‐way ANOVA with Tukey's post‐test. *P < 0.05, **P < 0.01, ***P < 0.001, ****P < 0.0001.

Having verified the efficient therapeutical efficacy in previous models through multiple doses, it is expected that a more robust therapeutical index can be achieved with minimal frequency of administration, even in large established tumors, which is a great challenge in the field of cancer immunotherapy. Given the photothermal conversion properties of Mn_3_O_4_,^[^ ^30^ ^]^ we next explored the inherent photothermal effect of Mn‐LDH on the eradication of established tumors in mice (**Figure** **7**A). For this, the tumor inhibition of Mn‐LDH against primary and distal tumors was investigated in a well‐established tumor model (initiated at a size of up to 120 mm^3^) using a diminished dosage and reduced administration frequency.

Initially, we assessed the photothermal conversion potential of Mn‐LDH both in vitro and in vivo. We first investigated the concentration‐dependent photothermal properties of Mn‐LDH in vitro (Figure 7B; Figure S29, Supporting Information). The maximum temperature exhibited no significant variation during the heating‐cooling cycle, indicating the good photothermal stability of Mn‐LDH (Figure 7C). Balancing the need for effective treatment with safety considerations, we aimed to control the temperature of tumor tissue below 50 °C after laser exposure, achieved by controlling the laser intensity or Mn‐LDH concentration. A moderate heat at ≈45 °C is considered to maintain safety while facilitating immunological responses.^[^ ^31^ ^]^ As anticipated, following i.t. injection of therapeutically equivalent Mn‐LDH, the central temperature of the tumor area rapidly reached 44.9, 46.8, and 47.3 °C after 1, 3, and 5 min of 808 nm irradiation with a power density of 1.0 W cm^−2^ (Figure 7D). In contrast, the temperature around the tumor area in mice injected with PBS remained in the range of 34.2–35.9 °C. As depicted in Figure 7E, following a 6 h incubation of Mn‐LDH (60 µg mL^−1^ manganese equivalent) with 4T1 cells, a majority of the 4T1 cells remained viable (green fluorescent‐labeled). Subjecting to a 10‐min exposure to 808 nm laser irradiation with a power density of 1.5 W cm^−2^, a significant portion of the 4T1 cells exhibited red fluorescence, indicative of cell membrane damage.

As illustrated in Figure 7F, 4T1 tumors were implanted in mice on days −9 and −3 before administration, with administration and laser irradiation into the primary tumor with a volume at 120 mm^3^. Mice treated with Mn‐LDH (without irradiation) displayed ≈30.7% inhibition of primary tumor, which increased to 90.0% after irradiation, as depicted in Figure 7G,I and Figure S30A (Supporting Information). Additionally, when combined with anti‐PD‐1 antibodies, Mn‐LDH treatment resulted in 60.0% of primary tumors free. Similar effects were observed in the growth inhibition of distal tumors, as shown in Figure 7H,J and Figure S30B (Supporting Information). The volume of distal tumors in mice treated with PBS, PD‐1, Mn‐LDH, Mn‐LDH+NIR, Mn‐LDH+NIR+PD‐1 was 165.4, 121.5, 71.2, 43.1, 28.3 mm^3^, respectively. This effect was further enhanced through the combined treatment with anti‐PD‐1 antibodies. The substantial tumor‐associated antigens (TAAs) exposure following tumor ablation further stimulated immune responses under moderate photothermal heating (≈45 °C).^[^ ^31^ ^]^ In this sense, a variety of innate and adaptive immune cells were recruited and mobilized to eliminate large tumor burdens and distal tumors. Collectively, the inherent photothermal effect of Mn‐LDH further facilitates the immune response, achieving superior therapeutical efficacy in the eradication of large established tumors of 4T1 TNBC with a single treatment. To our best knowledge, tumors of this size have not previously been curable by treatments that rely on endogenous immunity.

## 3 Conclusion

In summary, we have developed an engineered manganese‐doped layered double hydroxide (Mn‐LDH) through a straightforward hydrothermal method to reshape TME for cancer immunotherapy. The LDH effectively neutralized protons, supplemented Mg^2+^ for T cell activation, and the inclusion of Mn_3_O_4_ rendered LDH with cGAS‐STING activation, oxygen generation, and efficient photothermal effect, collectively demonstrating a robust TME reprogramming capability that transformed the immunosuppressive TME into an immune‐supportive one. Crucially, we discovered that the combination of Mn^2+^ and Mg^2+^ initiates a robust cascade immune response, both collaboratively promote the innate and adaptive immunity by activating the cGAS‐STING signaling pathway and sustaining the cytotoxicity of CD8^+^ T cells, respectively. Importantly, Mn‐LDH achieved superior therapeutical efficacy in the eradication of large established tumors with a single administration and photo‐irradiation. Collectively, these results indicated that Mn‐LDH could serve as a versatile platform for localized drug delivery, enabling multidimensional regulation of the TME and triggering a cascade of immune activation to potentiate metalloimmunotherapy. It may guide the rational design of cascade‐like metal‐based immunotherapy drugs that reshape suppressive TME comprehensively for efficient cancer immunotherapy.

## 4 Experimental Section

### Reagents and Chemicals

NaOH, MnCl_2_·4H_2_O, and NaCl were purchased from Macklin (Shanghai, China). MgCl_2_ was purchased from Innochem (Beijing, China). GSH and AlCl_3_ were purchased from Aladdin (LA, USA). BSA and 2‐(4‐Amidinophenyl)−6‐indolecarbamidine dihydrochloride (DAPI) were purchased from Solarbio (Beijing, China). GM‐CSF and IL‐4 were purchased from Pepro Tech (Cranbury, USA). All antibodies used for flow cytometry were purchased from Invitrogen (Carlsbad, USA). All ELISA kits were purchased from Elabscience (Wuhan, China). Calcein/PI Cytotoxicity Assay Kit and BCA Protein Assay Kit were obtained from Beyotime (Shanghai, China).

### Synthesis of LDH and Mn‐LDH

Mn‐LDH was synthesized according to the previously reported method with modification.^[^ ^32^ ^]^ Briefly, NaCl (275.3 mg) was added to 40 mL of a mixed aqueous solution containing MgCl_2_ (0.02 m), MnCl_2_·4H_2_O (0.01 m) under constant stirring (500 rpm) at 65 °C. Then, 35 mL of 0.25 m NaOH was added to the mixed aqueous solution with stirring (500 rpm) at 65 °C at constant temperature. After a reaction for 20 h, the collected Mn‐LDH was centrifugated at 8000 rpm for 10 min and then washed 3 times with water at 12 000 rpm for 15 min. Mn‐LDH is eventually suspended in deionized water and stored in the refrigerator at 4 °C. LDH (MgAl‐LDH), which is composed of MgCl_2_ (0.03 m), and AlCl_3_ (0.01 m) was synthesized using the same method.

### Characterization of LDH and Mn‐LDH

The ions content (Mn^2+^, Mg^2+^, and Al^3+^) of LDH and Mn‐LDH were measured by Inductively Coupled Plasma Mass Spectrometry (ICP‐MS). The hydrodynamic size and zeta potential of Mn‐LDH and LDH were measured with an instrument of DLS. TEM images were captured using JEM‐2100F (JEOL). Powder XRD patterns and XPS spectra of Mn‐LDH and LDH were measured.

### Mg^2+^ and Mn^2+^ release from Mn‐LDH

Mn‐LDH with a concentration of 200 µg mL^−1^ was dispersed in 50 mL of PBS (pH 6.5) with or without 1 mm of H_2_O_2_ under 500 rpm of stirring. At a predetermined time point, 1 ml of the buffer was taken out and the Mn‐LDH without degradation was removed by centrifugation (12 000 rpm for 15 min). The concentration of Mg^2+^ and Mn^2+^ in the supernatant was detected by ICP‐MS.

### Detection of Solution pH In Vitro

The pH of Mn‐LDH‐containing aqueous solution and Mn‐LDH‐containing PBS buffer were detected utilizing a pH meter. To identify the acid resistance of LDH to tumor cells, 4T1 cells were seeded in a 24‐well plate (10^5^ cells per well) and cultured in 1 mL RPMI 1640 culture medium overnight. The pH of the cell‐free, LDH‐ or Mn‐LDH‐containing culture medium was detected every 12 h for 2 days using the same pH meter.

### Detection of Tumor Tissue *pH* In Vivo

24 h after i.t. administered with PBS, LDH (25 mg kg^−1^), and Mn‐LDH (25 mg kg^−1^), the acidity neutralization ability of Mn‐LDH in vivo was investigated using a pH microelectrode. The pH value of each tumor was averaged over 3 measurements.

### O_2_ Generation Detection

Different concentrations of H_2_O_2_ were added to 5 mL aqueous solution with pre‐determined concentrations of Mn‐LDH, then the contents of dissolved O_2_ were measured by a dissolved oxygen meter for 20 min. For in vivo detection of hypoxia conditions in tumor tissue, PA was performed to monitor the vascular saturated oxygen in tumor tissues. HbO_2_ was detected at an excitation wavelength of 850 nm and Hb at 700 nm using a preclinical photoacoustic computerized tomography scanner 24 h after i.t. injected 50 µL PBS, LDH (25 mg kg^−1^), and Mn‐LDH (25 mg kg^−1^).

### Cellular Uptake Assessment of Mn‐LDH

To prepare fluorescently labeled Mn‐LDH, BSA‐Cy5 was first synthesized by a simple one‐step reaction between N‐hydroxysuccinimide (NHS)‐activated Cy5 and BSA. 0.5 mg of BSA‐Cy5 was added to 2 mL of an aqueous solution containing 1 mg Mn‐LDH with 500 rpm stirring at room temperature. After stirring for 40 min, BSA‐Cy5‐labeled Mn‐LDH was obtained and then washed 3 times at 12 000 rpm for 15 min. Then, 50 µg mL^−1^ BSA‐Cy5‐labeled Mn‐LDH was co‐cultured with DC2.4 cells which were seeded in a 24‐well plate (8 × 10^4^ cells per well) overnight. At a predetermined time point, the cells were washed with PBS and then stained with DAPI. A fluorescence microscope CKX53 (Olympus) was used to observe endocytosis by DC2.4 cells. The mean fluorescence intensity of DC2.4 cells was measured by flow cytometry.

### cGAS‐STING Activation Assay

To determine whether Mn‐LDH demonstrated a superior STING activation compared with free Mn^2+^, 32, 48, and 80 µg mL^−1^ of Mn‐LDH (the equivalent of 4, 6, and 10 µg mL^−1^ of Mn^2+^, respectively) were cocultured with RAW‐Lucia ISG cells which were seeded in a 96‐well plate (2 × 10^5^ cells per well) overnight. The Lucia luciferase detection reagent QUANTI‐Luc was performed to evaluate the activation of the cGAS‐STING signaling pathway at a predetermined time point. This assay was used to compare the activation of STING pathway among different reagents (Mg^2+^: 6.15 µg mL^−1^, LDH: 50 µg mL^−1^, Mg^2+^: 6.2 µg mL^−1^, Ions (namely 6.15 µg mL^−1^ Mg^2+^ and 6.2 µg mL^−1^ Mn^2+^, and Mn‐LDH: 50 µg mL^−1^). Next, the activation of downstream signaling pathways of STING was determined on BMDCs by western blot. Typically, different reagents (Mg^2+^: 6.15 µg mL^−1^, LDH: 50 µg mL^−1^, Mg^2+^: 6.2 µg mL^−1^, Ions (namely 6.15 µg mL^−1^ Mg^2+^ and 6.2 µg mL^−1^ Mn^2+^, and Mn‐LDH: 50 µg mL^−1^) were co‐cultured with BMDCs (6‐well plate, 4 × 10^6^ cells per well) for 24 h. Subsequently, proteins were lysed with RIPA lysate, separated by SDS polyacrylamide gel electrophoresis, and then transferred to a polyvinylidene fluoride membrane. The membrane was blocked with TBST solution containing 5% skim milk powder for 2 h at room temperature and incubated with primary antibodies for 1 h at room temperature, including p‐TBK1, p‐STING, p‐NF‐κB p65, and β‐actin rabbit antibodies. The goat anti‐rabbit IgG second antibody was added, and the solution was incubated for another 1 h at room temperature. Eventually, ECL substrate western blotting was performed to visualize protein bands by chemiluminescence detection.

### Cultivation of BMDCs and Maturation Assay

BMDCs were harvested to detect the relevant immune indicators in vitro. To be specific, bone marrow cells that derived from the femur and tibia of mice were cultured with an RPMI 1640 medium containing GM‐CSF (20 ng mL^−1^) and IL‐4 (10 ng mL^−1^). The culture medium was refreshed every 2 days and cells were collected on the 6th day for the maturation assay. To evaluate the maturation of BMDCs, the cells were seeded in a 24‐well plate (3 × 10^5^ cells per well) and cultured in 1 mL RPMI 1640 medium overnight. BMDCs were then co‐cultured with different reagents on 24‐well plates (Mg^2+^: 6.15 µg mL^−1^, LDH: 50 µg mL^−1^, Mg^2+^: 6.2 µg mL^−1^, Ions (namely 6.15 µg mL^−1^ Mg^2+^ and 6.2 µg mL^−1^ Mn^2+^), and Mn‐LDH: 50 µg mL^−1^) for 48 h and stained with anti‐CD40, anti‐CD80, anti‐CD86, and anti‐CD11c antibodies. Then, a flow cytometer (Invitrogen Attune NxT) was performed to detect the expression of co‐stimulatory molecules CD40, CD80, and CD86. ELISA kit was used to measure the secretion level of IFN‐β in the supernatant of the cultural medium.

### Biosafety Analysis of Mn‐LDH

All animal studies were in accordance with the guidelines approved by the animal care and use committee at Peking Union Medical College, China (Approval number: 1 121 110 500 028). 6 mice were divided into 2 groups randomly and then were i.t. administrated with PBS, Mn‐LDH (25 mg kg^−1^) every 4 days. The body weight of each mouse was detected every 2 days. On day 16, blood was collected from the ocular venous plexus of mice for subsequent assay of liver and kidney function as well as hemolysis. For the hemolysis assay, a whole blood sample was taken in an anticoagulant tube and centrifuged at 3000 rpm for 15 min to obtain red blood cells. The red blood cells were re‐suspended with PBS to obtain 2%−4% red blood cell suspension. The suspension was mixed with Mn‐LDH of different concentrations and incubated at 37 °C for 2 h to determine the absorbance at 570 nm and calculate the hemolysis rate.

### In Vivo Anti‐Cancer Effect of Mn‐LDH and Immunological Analysis

To construct subcutaneous breast tumor models, 5 × 10^5^ 4T1 cells were injected into 6–8 weeks old female BALB/c mice. 24 mice were divided into 4 groups randomly and then were i.t. administrated with PBS, LDH, Ions, and Mn‐LDH (n = 6, LDH: 25 mg kg^−1^, Ions: 3.08 mg kg^−1^ of Mg^2+^ and 3.10 mg kg^−1^ of Mn^2+^, Mn‐LDH: 25 mg kg^−1^) at predetermined time. The tumor volume and weight of mice were measured every 2 days. 21 days after tumor implantation, 5 mice were sacrificed, and then tumors, spleen, and LNs were stripped for the subsequent analysis of immune cells. The remaining 1 mouse of each group was dissected with heart, liver, spleen, lungs, and kidneys for H&E staining. Next, the stripped tumors were cut into 2 pieces for subsequent immunofluorescent staining and immune cell analysis. Cells were stained with fluorescence‐labeled antibodies of F4/80, CD80, CD206, CD3, CD4, CD8, IFN‐γ, and Granzyme B to analyze the level of TAMs and T cells in the tumor. The isolated tumor supernatant was then used to measure the secretion level of IFN‐β and CXCL‐10. The cells in LNs were stained by fluorescence‐labeled antibodies of CD11c, CD80, CD86, CD3, CD4, and CD8 to analyze the level of DCs and T cells in LNs. Finally, immunofluorescence staining in tumor tissues for p‐STING^+^ and HIF‐1α^+^ cells was conducted through staining with anti‐p‐STING and anti‐HIF‐1α.

### In Vivo Anti‐Colorectal Cancer and Distant Effects of Mn‐LDH and Immunological Analysis

To construct subcutaneous primary colorectal tumor models, 1.8 × 10^6^ CT26 cells were injected into the right back of 6–8 weeks old female BALB/c mice. Another 1.8 × 10^6^ CT26 cells were injected into the left back of mice to construct a secondary colorectal tumor on the day before administration. 18 mice were divided into 6 groups randomly and then were i.t. administrated with PBS, LDH, Ions, and Mn‐LDH (n = 8, Mg^2+^: 3.08 mg kg^−1^, LDH: 25 mg kg^−1^, Mn^2+^: 3.10 mg kg^−1^, Ions: 3.08 mg kg^‐1^ of Mg^2+^ and 3.10 mg kg^−1^ of Mn^2+^, Mn‐LDH: 25 mg kg^−1^) at predetermined time. 15 days after tumor implantation, all mice were sacrificed, and then tumors, spleen, and LNs were stripped for the subsequent analysis of immune cells. The stripped tumors were cut into 2 pieces for subsequent immune cell analysis. Cells were stained with fluorescence‐labeled antibodies of F4/80, CD11c, CD80, CD206, CD3, CD4, and CD8 to analyze the level of TAMs, DCs, and T cells in the tumor. The cells in LNs were stained by fluorescence‐labeled antibodies of CD11c, CD80, CD86, CD3, CD4, and CD8 to analyze the level of DCs and T cells in LNs. Splenocytes were also collected to be stained by CD3, CD4, and CD8 to analyze the level of antigen‐specific T cells. In a parallel experiment, 60 mice were divided into 6 groups randomly and then were i.t. administrated with the aforementioned reagents. The tumor volume and weight of mice were measured every 2 days. 21 days after tumor implantation, 5 mice in each group were sacrificed to photograph and weigh the primary and secondary tumors. The remaining 5 mice in each group continued to be tested for survival rate until day 35 of primary tumor implantation.

### Photothermal Conversion Capacity of Mn‐LDH

To investigate the photothermal performance of Mn‐LDH, different concentrations of Mn‐LDH (equivalently the contents of Mn were 60, 120, and 180 µg mL^−1^, respectively) placed in 1.5 mL tubes were irradiated with an 808 nm laser for 5 min with a power density of 1.5 W cm^−2^, with PBS and LDH as control. The highest temperature in the field of view was recorded by an infrared thermal camera once a minute. Similarly, Mn‐LDH with the same concentrations were irradiated for 4 cycles with an 808 nm laser on and off at a power density of 1.5 W cm^−2^. The heating and cooling curves were recorded by the same infrared thermal camera. Next, in vitro photothermal anti‐cancer effects were studied by a live and dead assay using Calcein/PI staining. 6 × 10^4^ 4T1 cells were first seeded in a 24‐well plate and cultured in 1 mL RPMI 1640 culture medium overnight. Then, PBS or Mn‐LDH (equivalently the content of Mn was 180 µg mL^−1^) were added into wells and then irradiated with an 808 nm laser for 10 min at a power density of 1.5 W cm^−2^. 6 hours after being treated with or without irradiation, fluorescence microscope images were taken according to the procedures of Cell Viability/Cytotoxicity Kit. To determine the in vivo photothermal performance and keep the central temperature of the tumor under 50 °C by adjusting the power density of irradiation, 4T1‐bearing mice were i.t. administrated PBS or 25 mg kg^−1^ Mn‐LDH. The tumor area was then irradiated with an 808 nm laser at a moderate power density (1.0 W cm^−2^) for 5 min. The real‐time temperature around the tumors was recorded by the same infrared thermal camera.

### Photo‐Enhanced Anti‐Cancer Efficacy and Abscopal Effects

As described before, 5 × 10^5^ 4T1 cells were injected into the right back of 6–8 weeks old female BALB/c mice. Another 5 × 10^5^ 4T1 cells were injected into the left back of mice to construct a secondary TNBC tumor 3 days before administration. 25 mice were divided into 5 groups randomly and then were i.t. administrated with PBS and Mn‐LDH and i.p. administrated with PD‐1 antibodies (n = 5, Mn‐LDH: 25 mg kg^−1^, PD‐1 antibodies: 8 mg kg^−1^) at predetermined time. The tumor volume and body weight of mice were measured every 2 days. All mice were sacrificed, and then tumors were collected on day 23 post‐tumor implantation. The heart, liver, spleen, lungs, and kidneys were dissected for H&E staining to analyze systemic safety.

### Statistical Analysis

Results are expressed as mean ± SD, using GraphPad 8 software for statistical analysis. The number of repetitions of each experiment is indicated on the corresponding legend. For assessment of differences, one‐way ANOVA with Tukey's multiple comparisons test was used. *P < 0.05, **P < 0.01, ***P < 0.001, and ****P < 0.0001 was considered a which was considered significant, moderately significant, highly significant, and much more highly significant, respectively.

## Conflict of Interest

The authors declare no conflict of interest.

## Supporting information

Supporting Information

## Data Availability

The data that support the findings of this study are available from the corresponding author upon reasonable request.
