# Supplementary material for: Multi‐Metallic Nanosheets Reshaping Immunosuppressive Tumor Microenvironment through Augmenting cGAS‐STING Innate Activation and Adaptive Immune Responses for Cancer Immunotherapy
Source: Adv Sci (Weinh). 2024 Aug 9;11(38):2403347. doi: 10.1002/advs.202403347 (PMC11481177; doi:10.1002/advs.202403347)
Supplement: Supplementary file 1 — Supporting Information [file ADVS-11-2403347-s001.docx]

Supporting Information

*Yuxuan Peng, Shuang Liang, Dan Liu, Kongshuo Ma, Kaiqing Yun, Mengli Zhou, Linna Hai, Mengdi Xu, Yiyang Chen, Zhaohui Wang**

Y. Peng, S. Liang, D. Liu, K. Ma, M. Zhou, K. Yun, L. Hai, M. Xu, Y. Chen, and Z. Wang

State Key Laboratory of Bioactive Substance and Function of Natural Medicines,

Institute of Materia Medica, Chinese Academy of Medical Sciences & Peking Union Medical College, Beijing 100050, China

Email: zhaohuiwang@imm.ac.cn

Y. Peng, S. Liang, D. Liu, K. Ma, M. Zhou, K. Yun, L. Hai, M. Xu, Y. Chen, and Z. Wang

Beijing Key Laboratory of Drug Delivery Technology and Novel Formulation,

Institute of Materia Medica, Chinese Academy of Medical Sciences & Peking Union Medical College, Beijing 100050, China

Table S1. Molar ratios of ions in LDH and Mn-LDH.

| Nanoparticles | Mn:Mg:Al (molar ratio) | Mn^2+^-substitution efficiency (mg of Mn^2+^ per mg of Mn-LDH) |
| --- | --- | --- |
| LDH | 0.00: 2.50: 1.00 | / |
| Mn-LDH | 1.23: 2.26: 1.00 | 0.124 |


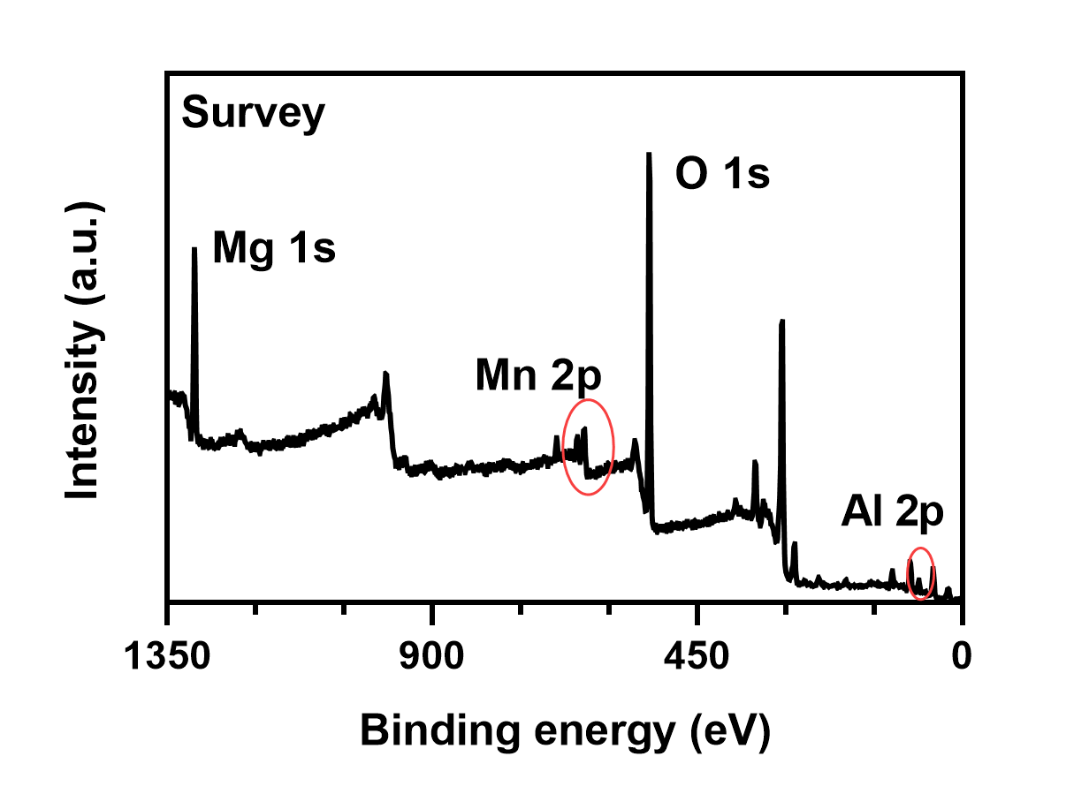


Figure S1. XPS pattern (survey) of Mg 1s, Mn 2p, O 1s, and Al 2p spectra of Mn-LDH.


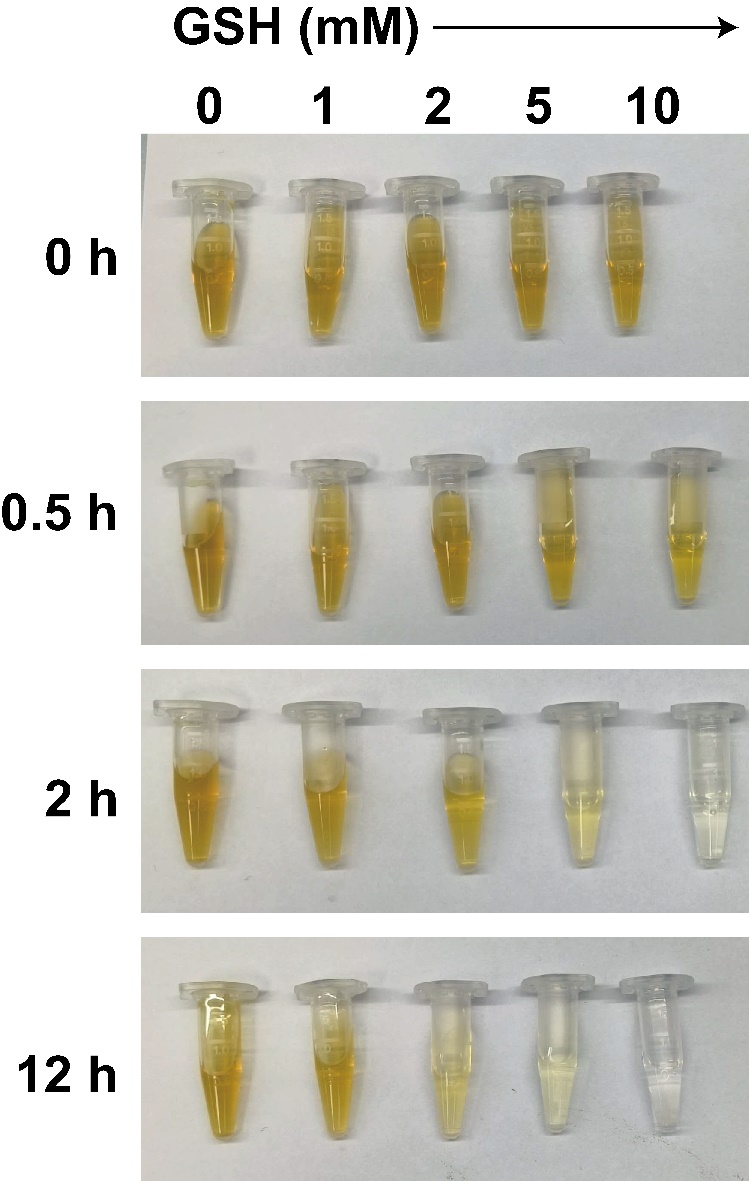


Figure S2. Photograph of Mn-LDH degradation in response to different concentrations of GSH in PBS buffer at the indicated time.


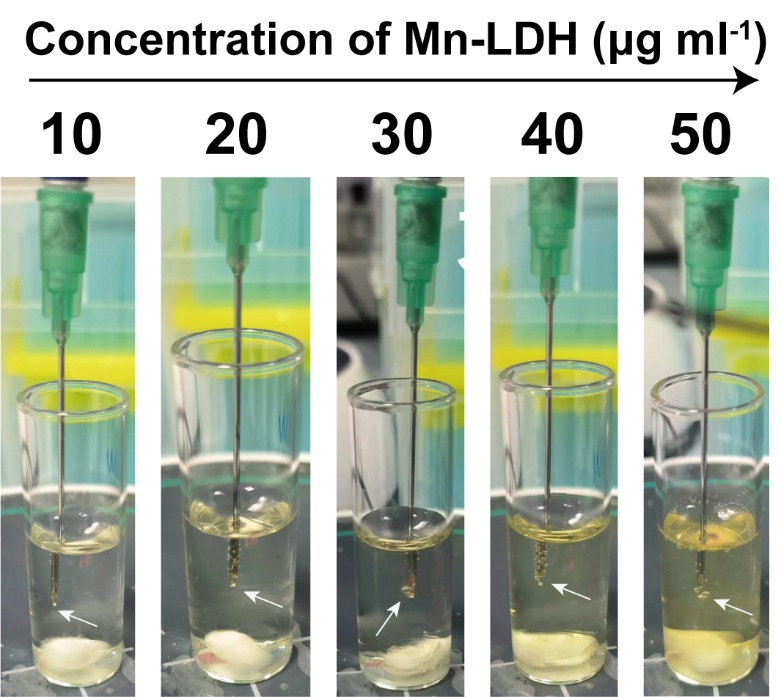


Figure S3. O_2_ generation of Mn-LDH at indicated concentration in the presence of 40 μM H_2_O_2_.


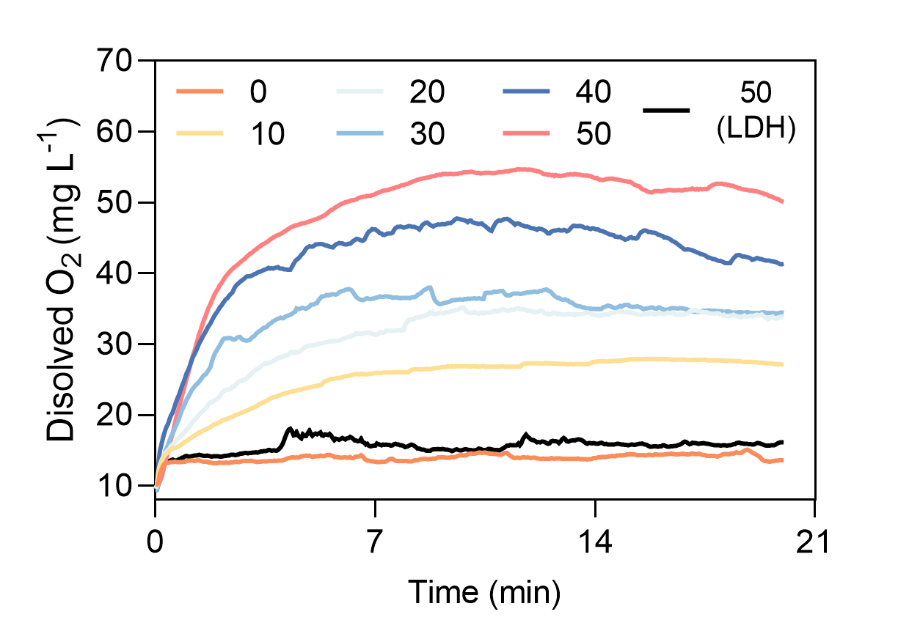


Figure S4. Oxygen generation of H_2_O_2_ (40 μM) treated by LDH (50 μg mL^−1^) and different concentrations of Mn-LDH.


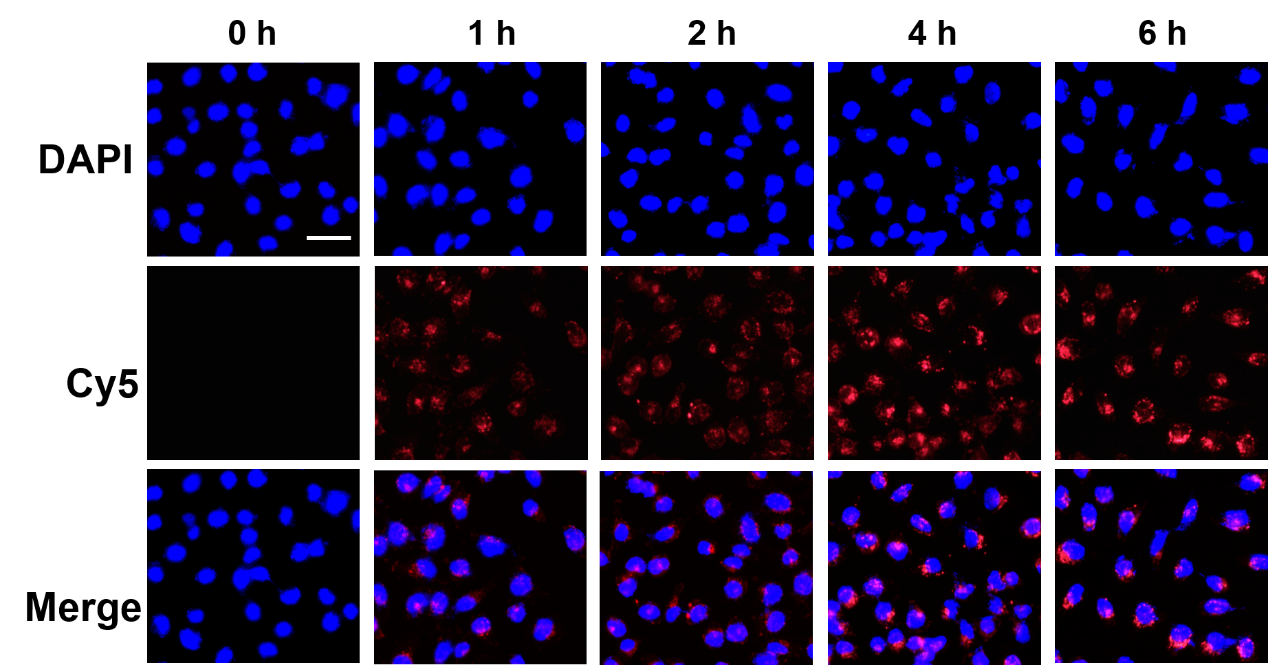


Figure S5. Fluorescence microscope images of DC2.4 cells that were treated with Mn-LDH. Red: BSA-Cy5-labeled Mn-LDH. Blue: nuclei (scale bar: 40 μm).


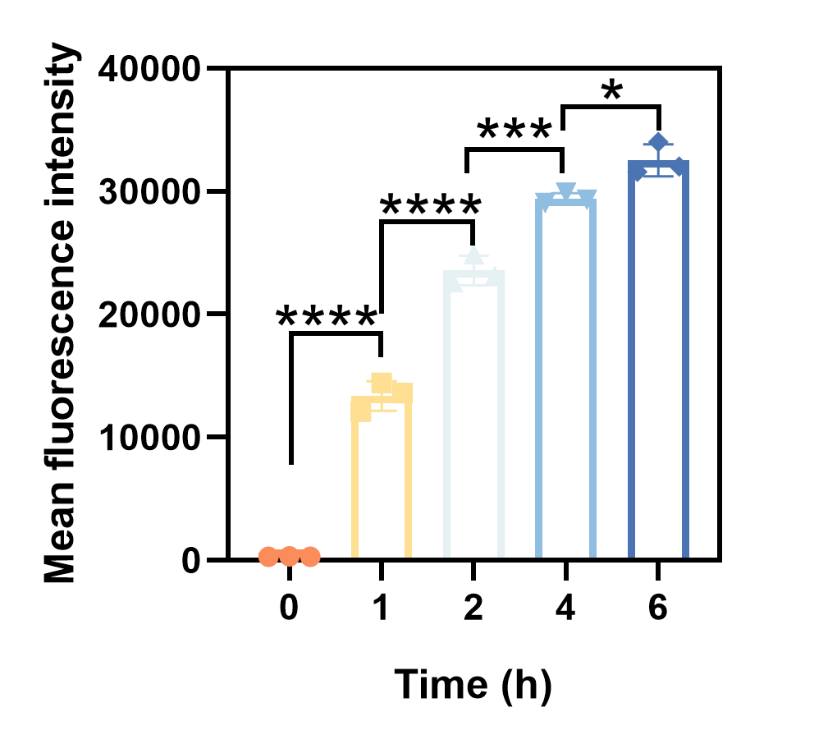


Figure S6. Mean fluorescence intensity (MFI) of Mn-LDH-treated DC2.4 cells at the indicated time (n = 3).


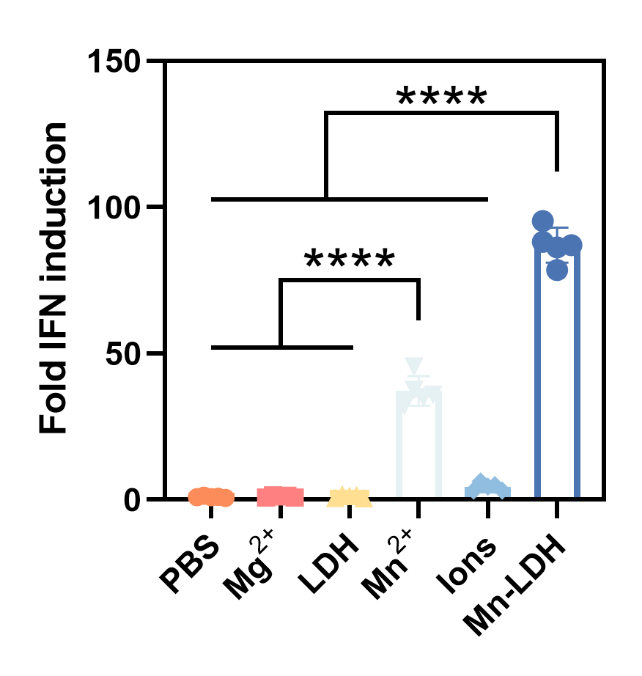


Figure S7. The expression of IFN-Ⅰ (normalized by PBS group) by RAW-Lucia ISG cells when treated with different agents 24 h later (n = 5).


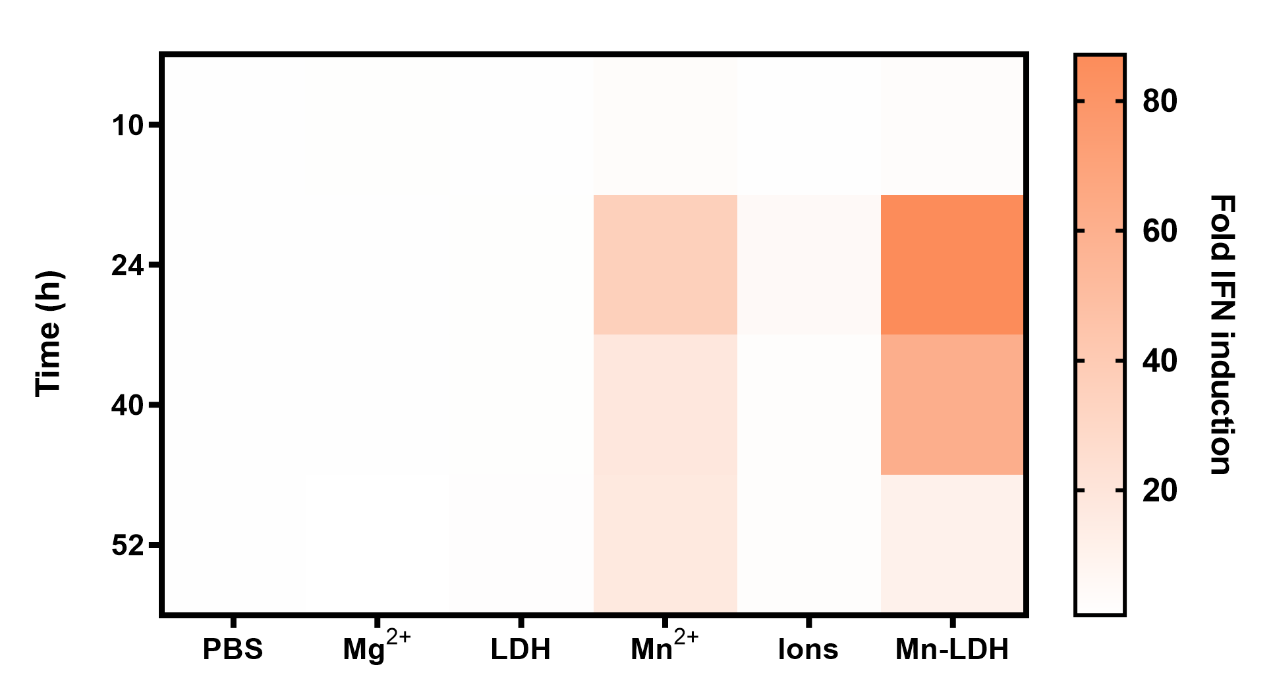


Figure S8. Heat map of the expression of IFN-Ⅰ (normalized by PBS group) by RAW-Lucia ISG cells when treated with different formulations (n = 5).


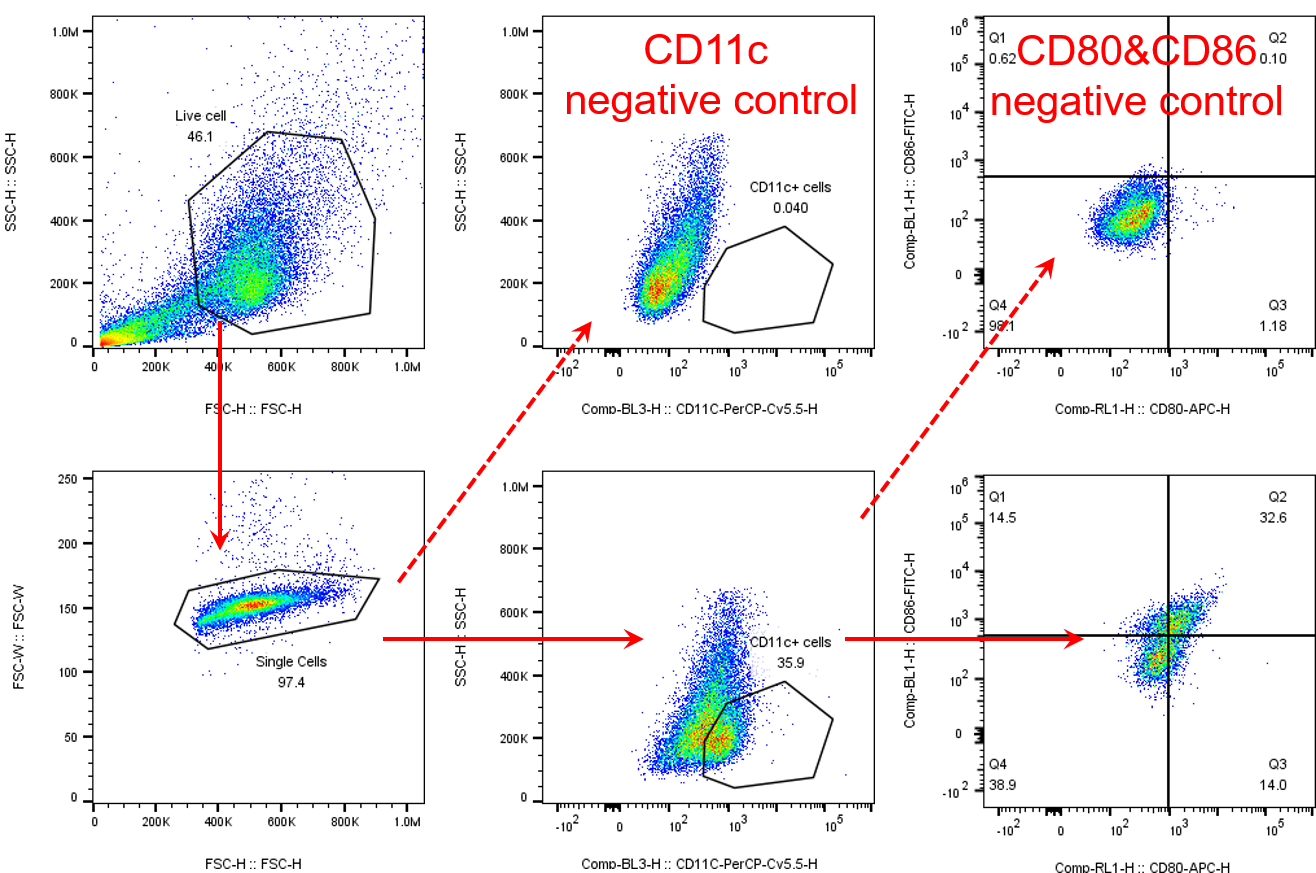


Figure S9. Circling gate scheme of flow cytometry of BMDCs. Gating single live cells according to size and granularity (FSC and SSC).  Circle the BMDCs according to CD11c. In the gate of “CD11c^+^ cells”, matured BMDCs (CD40^+^, CD80^+,^ and CD86^+^) were sorted out.


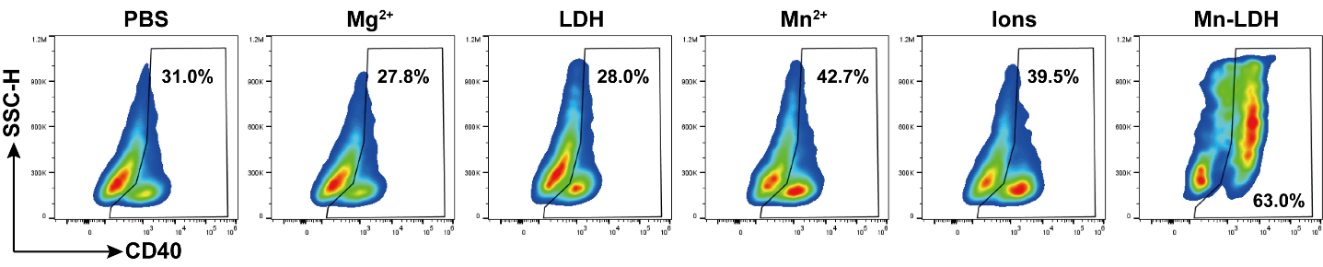


Figure S10. Representative FACS plots of CD40^+^ BMDCs after incubating with different reagents.


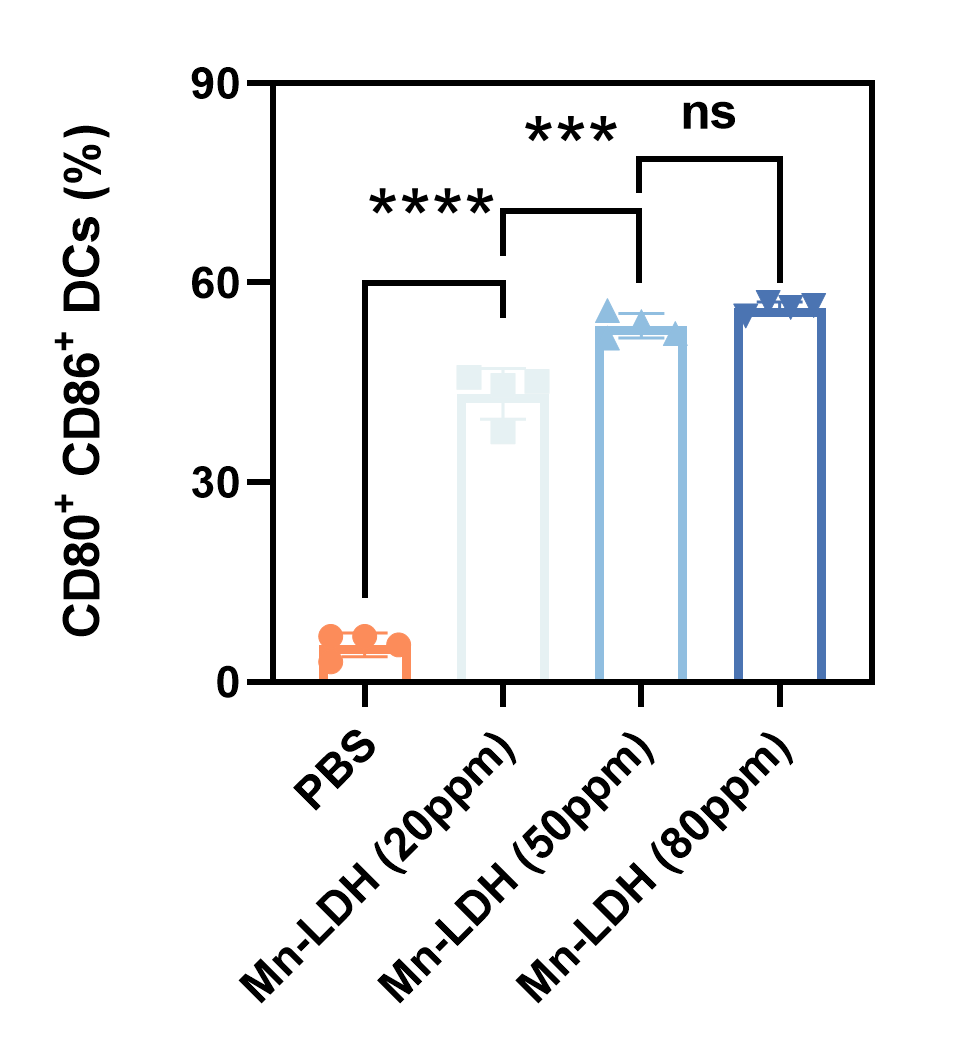


Figure S11. The proportion of CD80^+^ CD86^+^ BMDCs after incubating with different concentrations of Mn-LDH (n = 4).


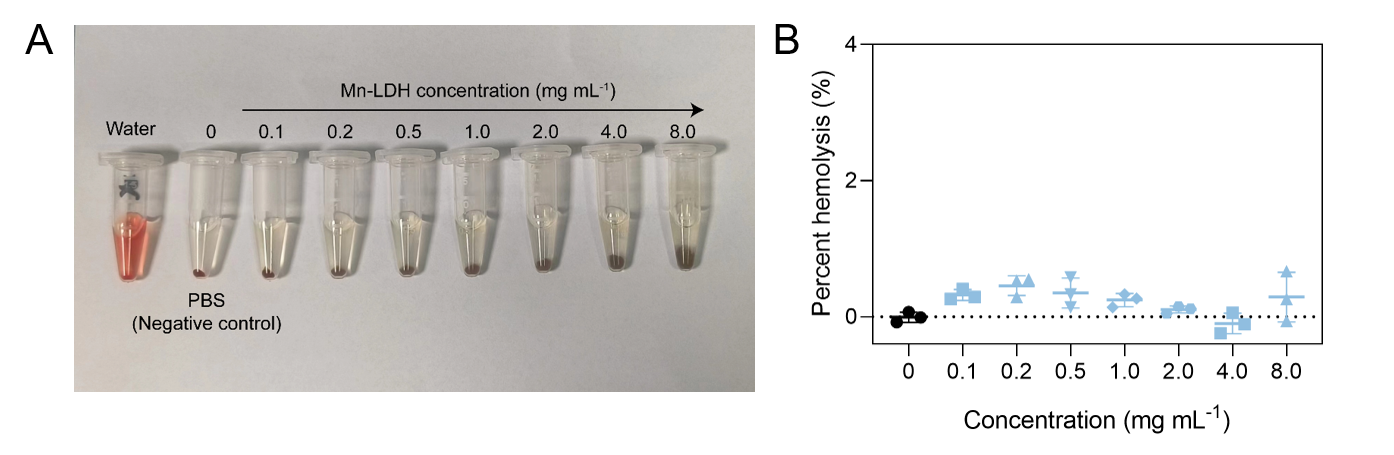


Figure S12. Photograph of hemolysis (A) and percent hemolysis (B) treated with different concentrations of Mn-LDH.


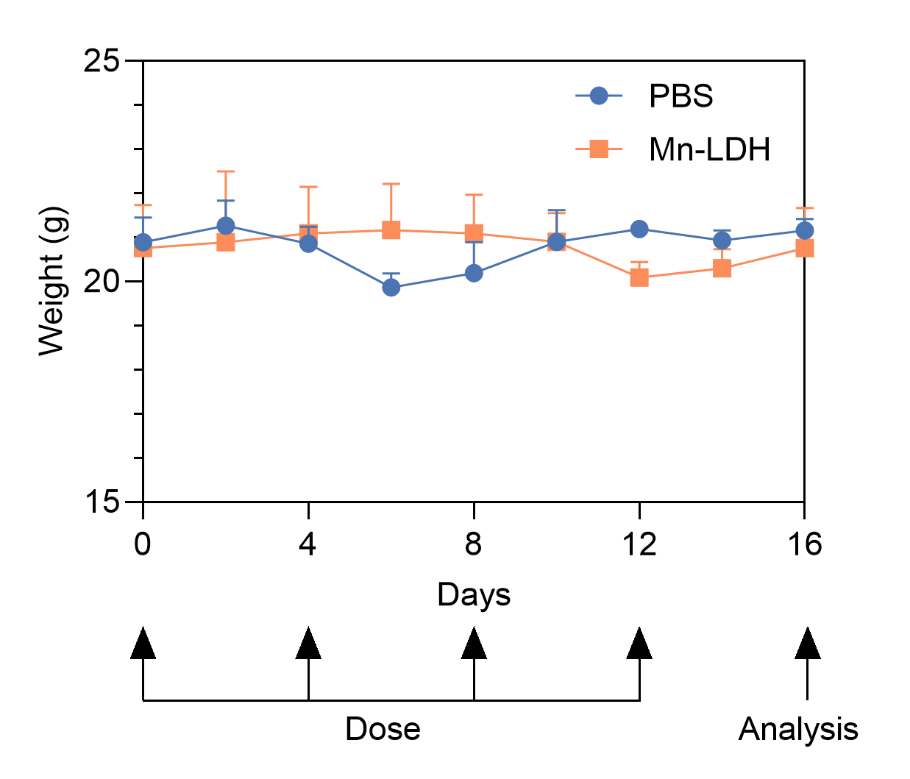


Figure S13. The body weight of healthy mice treated with PBS or Mn-LDH (25 mg kg^-1^) at the predetermined time (n = 3).


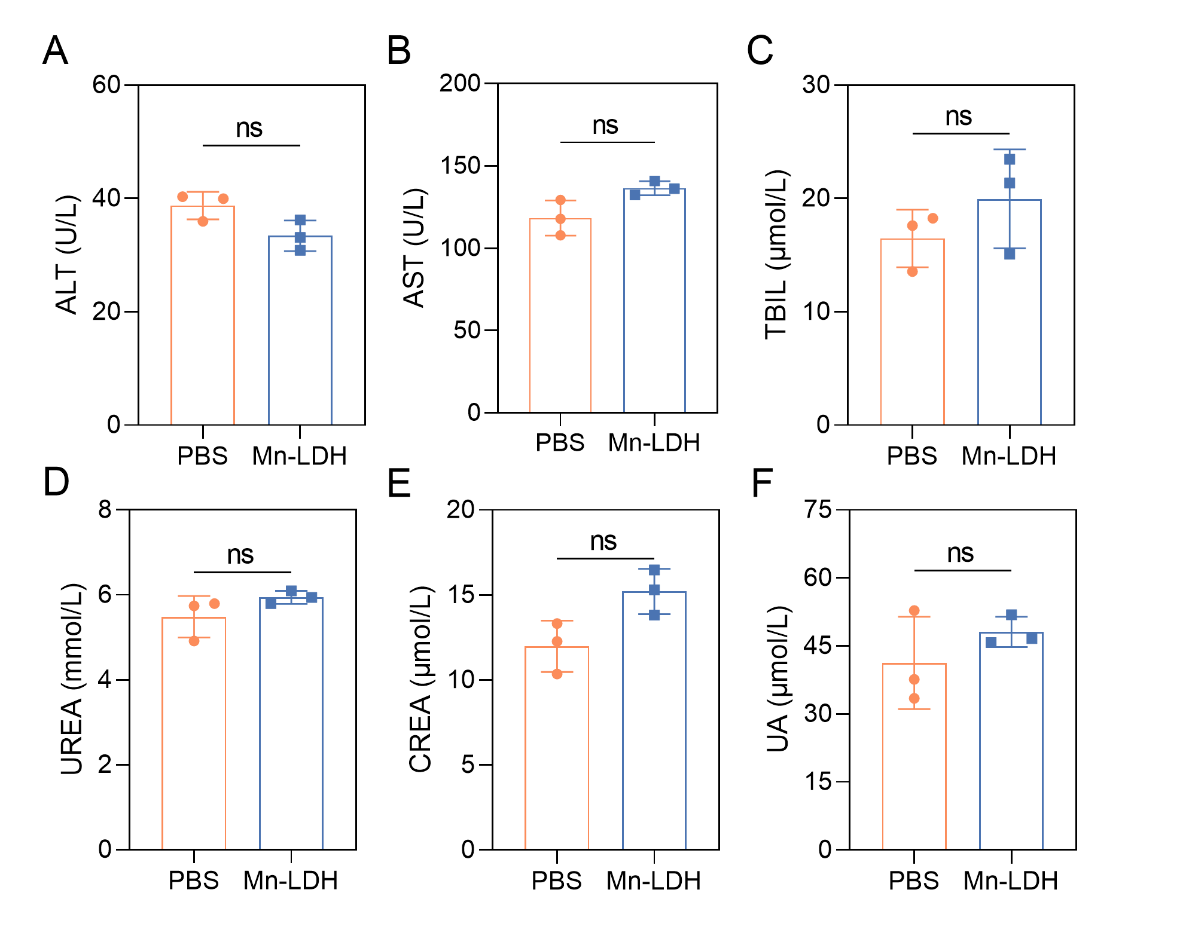


Figure S14. Hepatic and renal function indicators of PBS- or Mn-LDH-treated healthy mice (n = 3). (A-F) The concentration of alanine aminotransferase (ALT, A), Aspartate aminotransferase (AST, B), total bilirubin (TBIL, C), Urea (D), Crea (E) and Uricase (UA, F) in plasma.


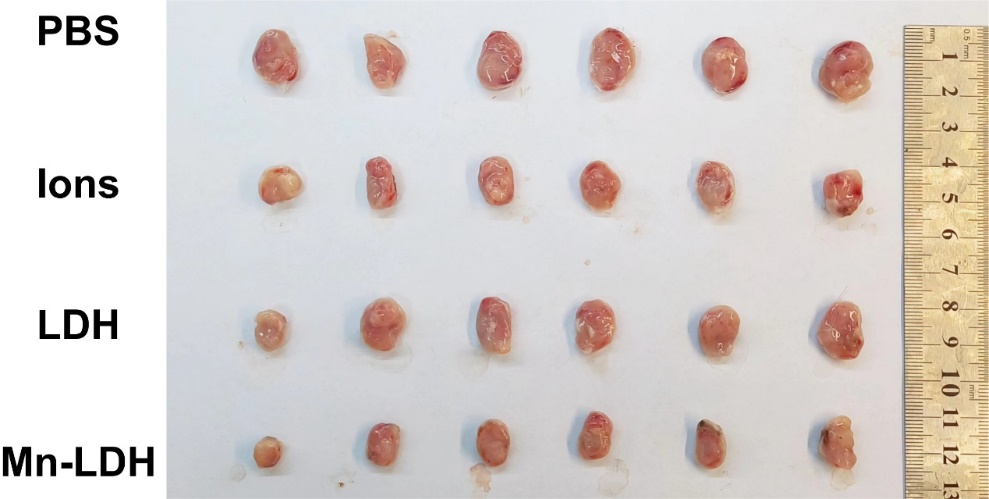


Figure S15. Photograph of excised 4T1 tumors (n = 6).


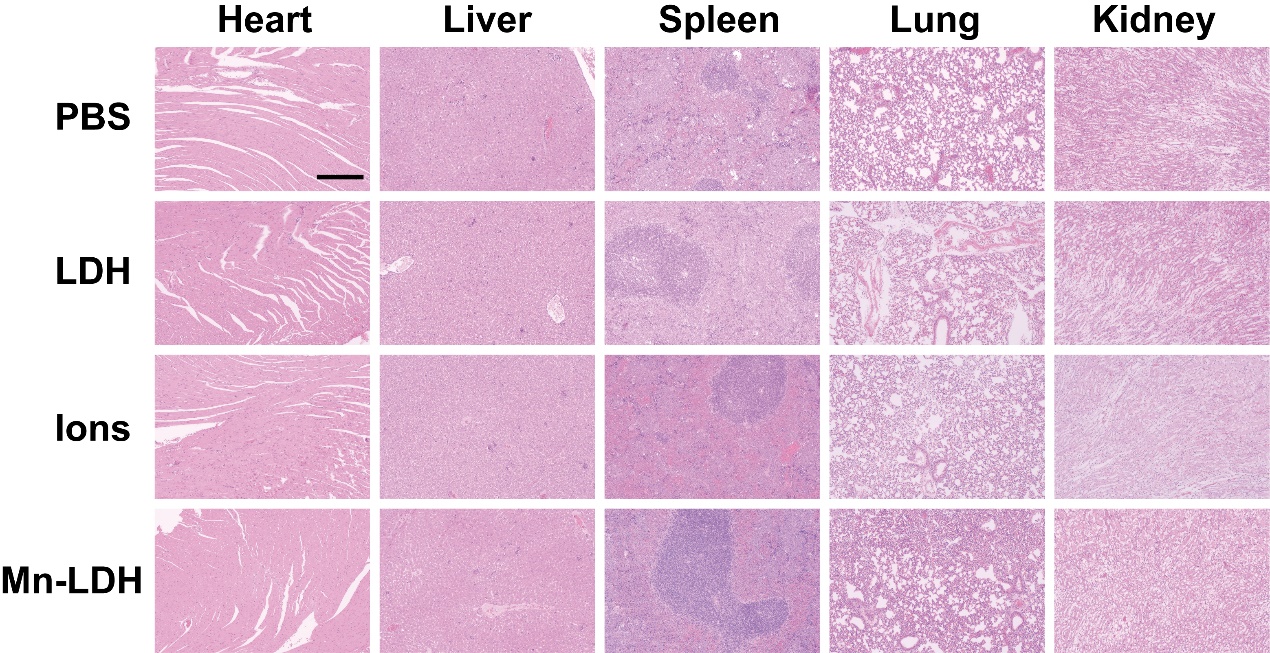


Figure S16. H&E-stained major organs of 4T1-bearing mice (Scale bar: 100 μm).


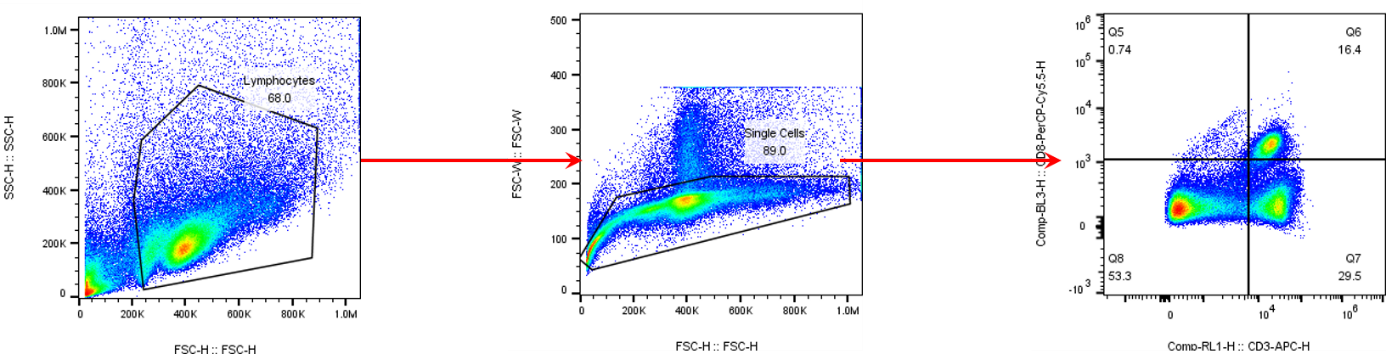


Figure S17. Circling gate scheme of cytotoxic T lymphocytes (CTLs) in LNs of 4T1-bearing mice. Gating single live cells according to size and granularity (FSC and SSC). In the gate of “single cells”, cytotoxic T lymphocytes (CD3^+^ and CD8^+^) were sorted out.


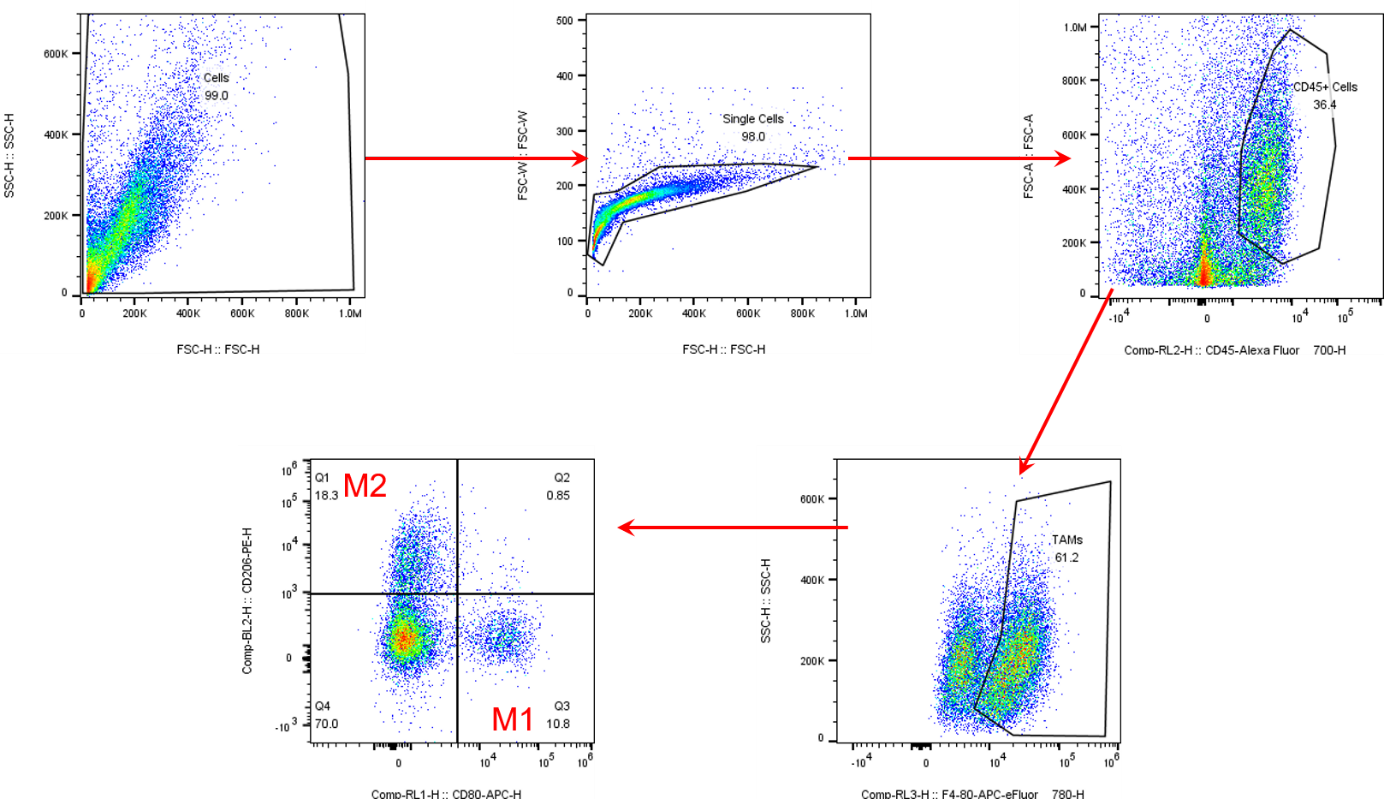


Figure S18. Circling gate scheme of TAMs in tumors of 4T1-bearing mice. Gating single live cells according to size and granularity (FSC and SSC). Circle the lymphocytes according to CD45. In the gate of “CD45^+^ cells”, TAMs (F4/80^+^) were sorted out. TAMs gate was divided into M1-TAMs (CD80^+^) and M2-TAMs (CD206^+^) subsets according to the expression of CD80 and CD206.


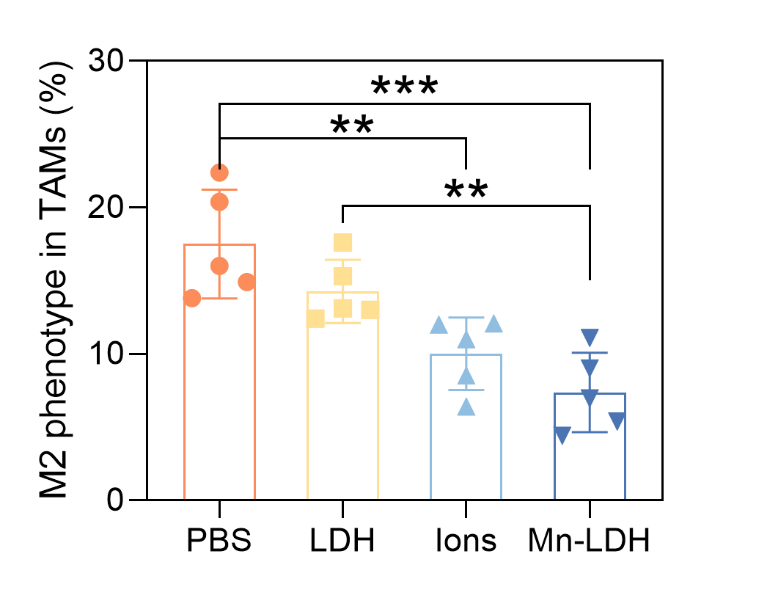


Figure S19. Quantitative analysis of the proportion of M2-phenotype TAMs (n = 5).


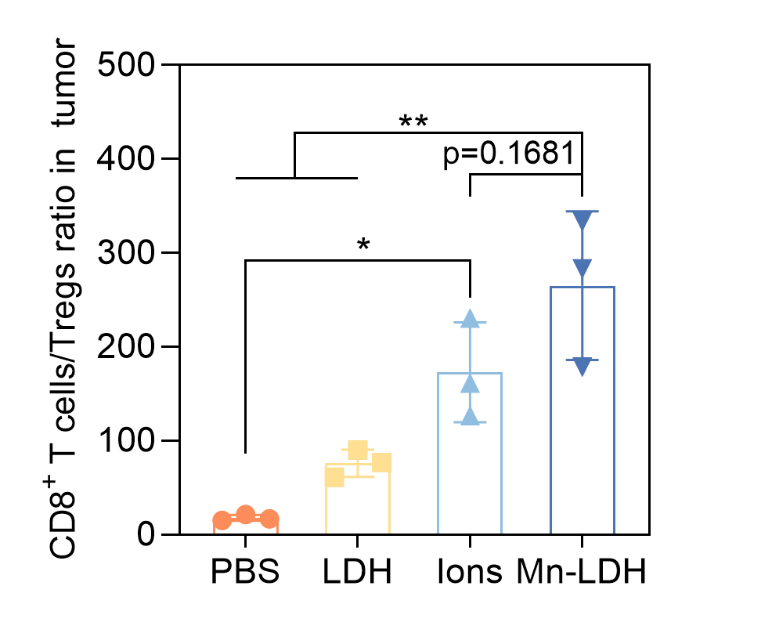


Figure S20. Quantitative analysis of CD8/Treg ratio in tumors of 4T1-bearing mice (n = 3).

**
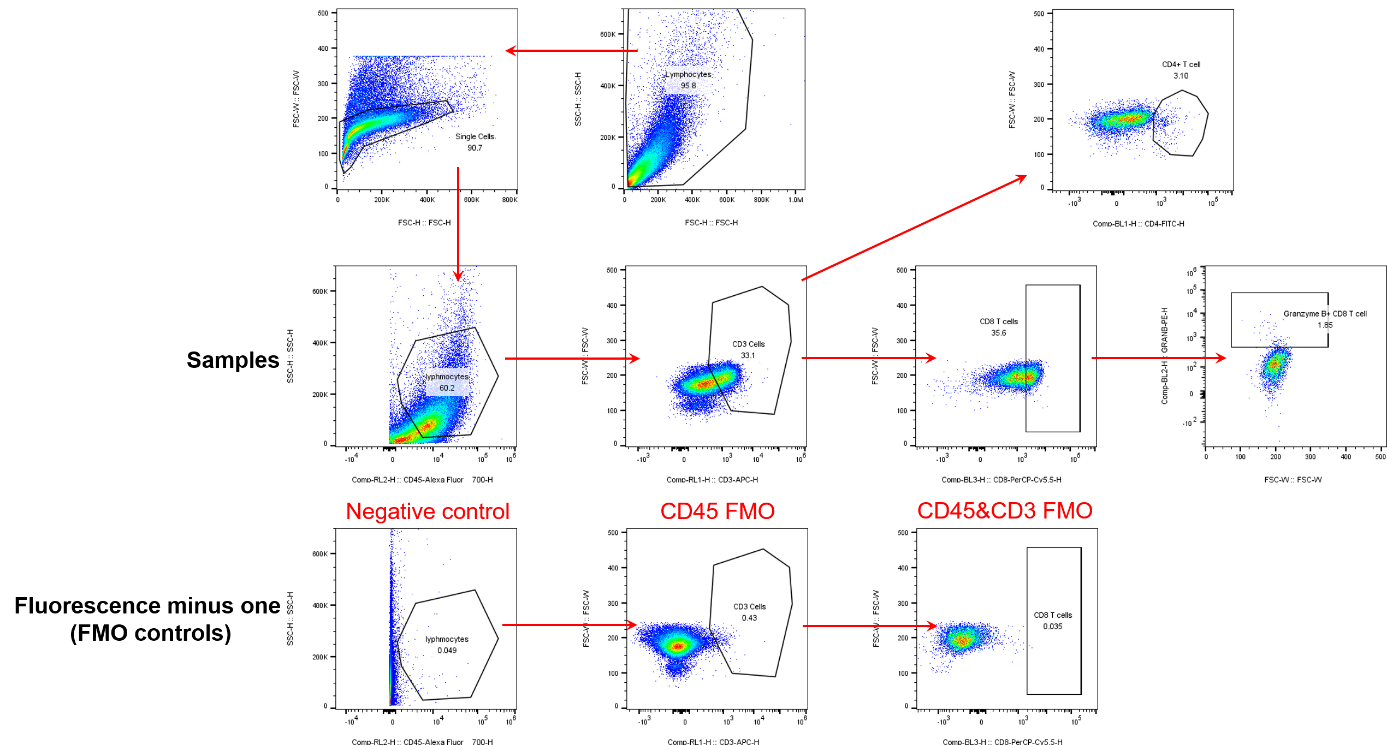
**

Figure S21. Circling gate scheme of flow cytometry of T cells in tumors of 4T1-bearing mice. Gating single live cells according to size and granularity (FSC and SSC). Circle the lymphocytes according to CD45. In the gate of “CD3^+^ cells”, T cells were sorted out. Helper T cells were sorted out according to the expression of CD4. Cytotoxic T lymphocytes were sorted out according to CD8, which was further divided into Granzyme B^+^ CTLs subsets according to the expression of Granzyme B.


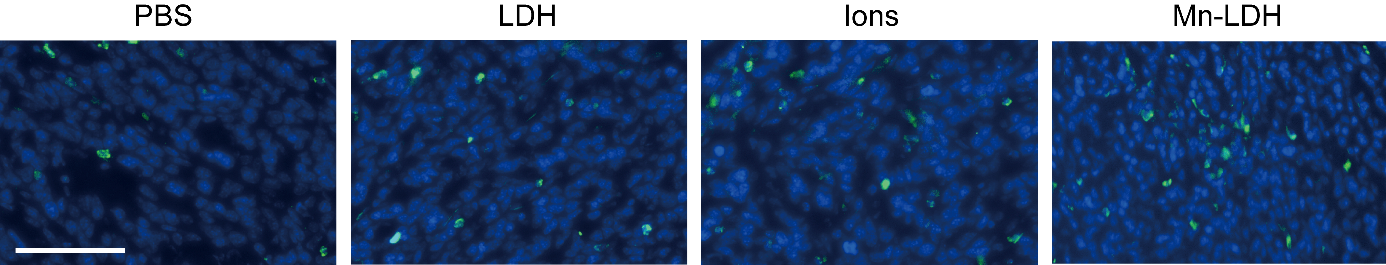


Figure S22. Immune fluorescence staining images of CD3^+^ cells in the frozen tumor section. Blue: nuclei. Green: FITC-labeled anti-CD3 antibodies (scale bar: 100 μm).


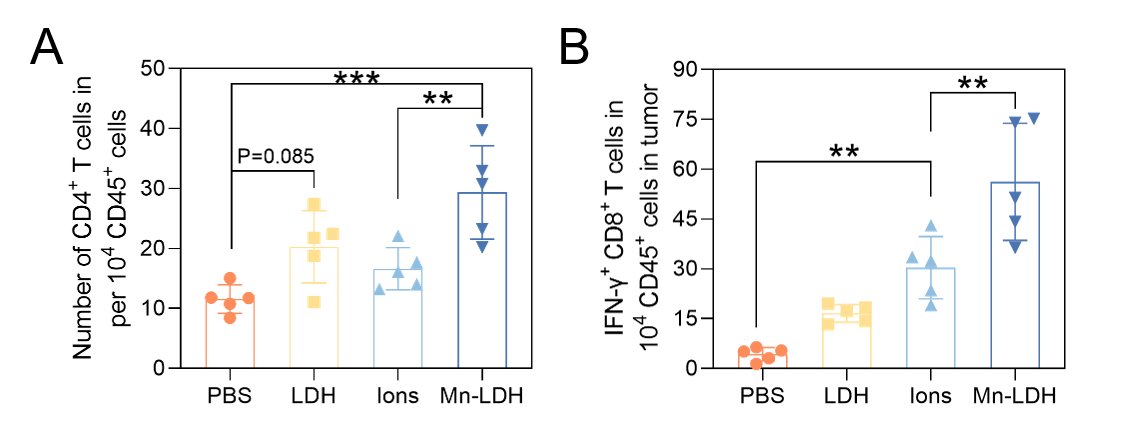

Figure S23. (A) Quantitative analysis of CD4^+^ T cells in CD45^+^ cells in tumor (n = 5). (B) Quantitative analysis of IFN-γ CD8^+^ T cells in CD45^+^ cells in tumor (n = 5).


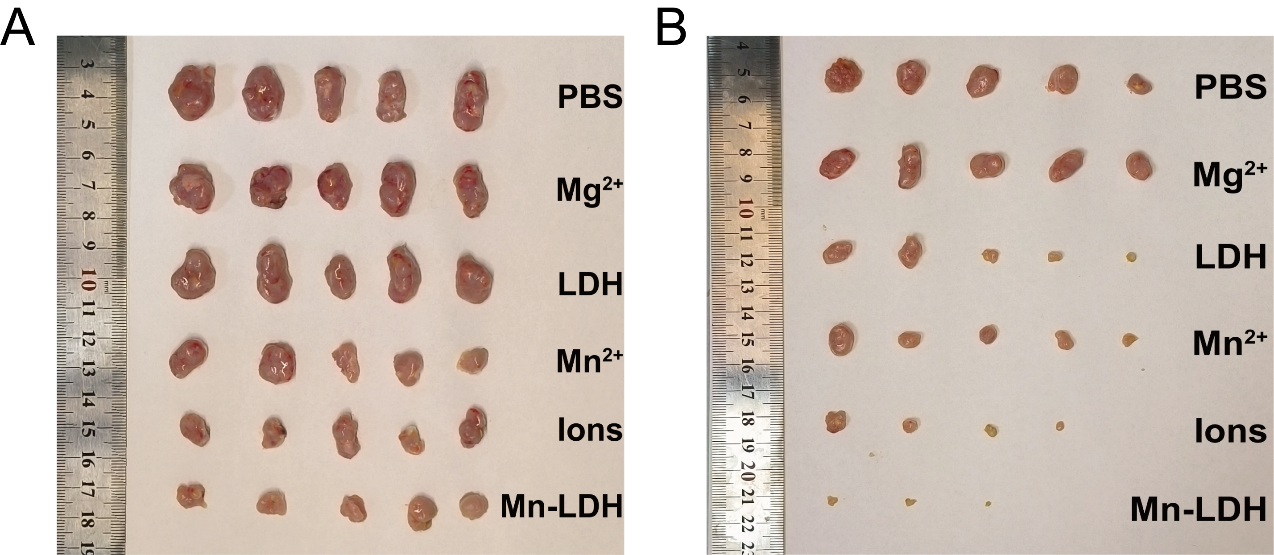


Figure S24. (A) Photograph of excised primary CT26 tumors. (B) Photograph of excised secondary CT26 tumors.


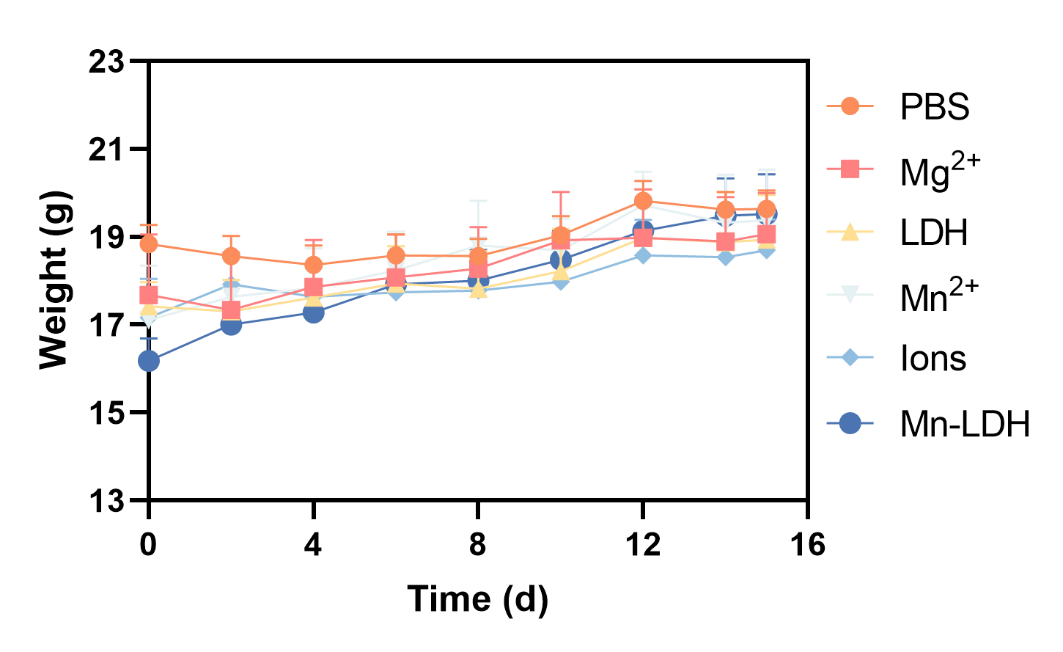


Figure S25. The body weights of CT26-bearing mice under different treatments at the indicated time.


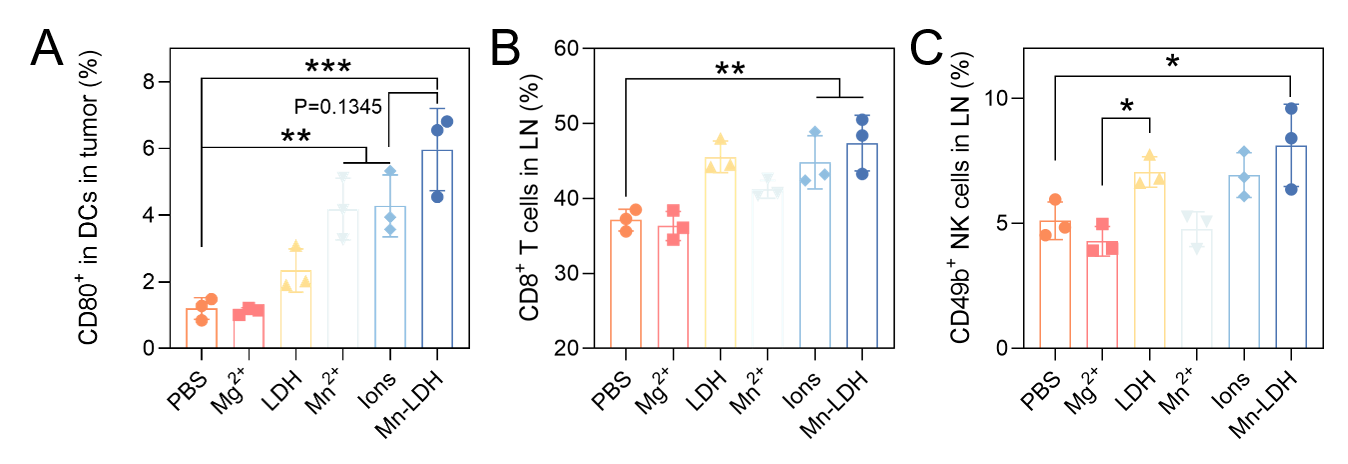


Figure S26. Quantitative analysis of the proportion of CD80^+^ DCs in CD11c^+^ cells (A), CD8^+^ cells in CD3^+^ cells in LNs (B), and NK cells in LNs of CT26-bearing mice (C).


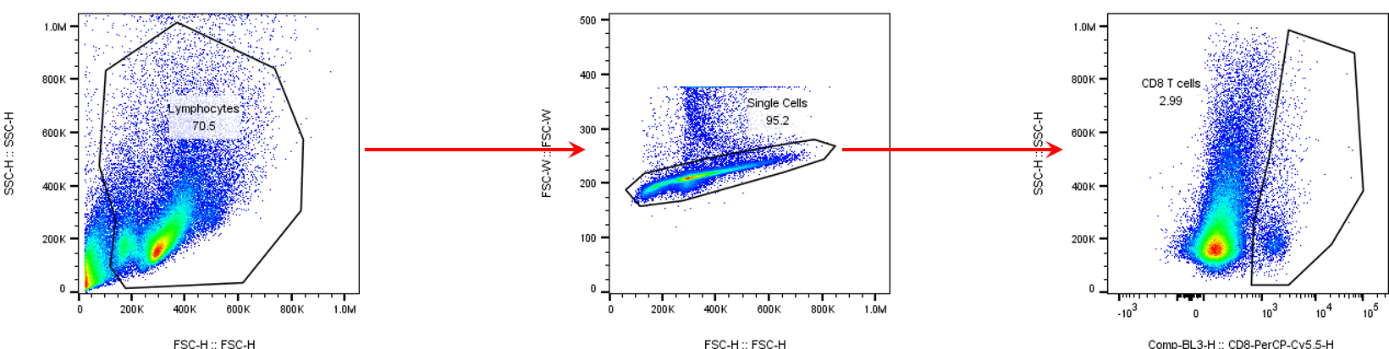


Figure S27. Circling gate analysis of CTLs in the spleen of CT26-bearing mice. Gating single live cells according to size and granularity (FSC and SSC). CTLs were sorted out according to CD8.

**
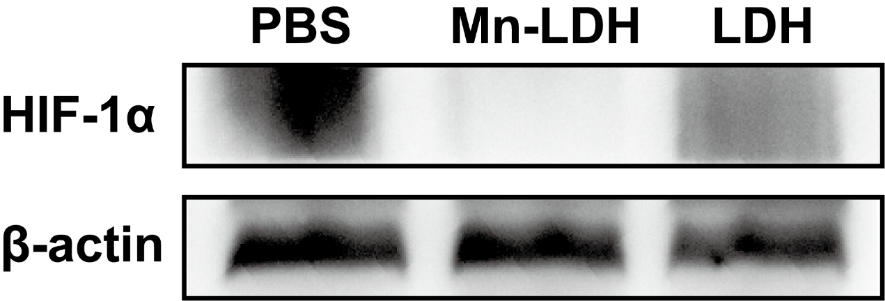
**

Figure S28. Western blot analysis of HIF-1α in CT26 tumor treated with different formulations.


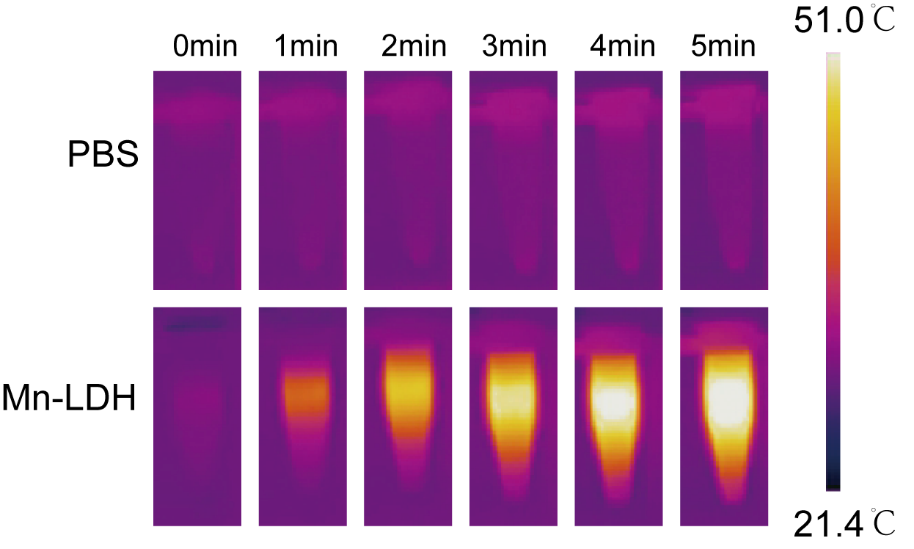


Figure S29. Infrared thermal images of test tubes containing or not containing Mn-LDH ([Mn] = 180 μg mL^-1^) under laser irradiation for 5 min (1.0 W cm^-2^).


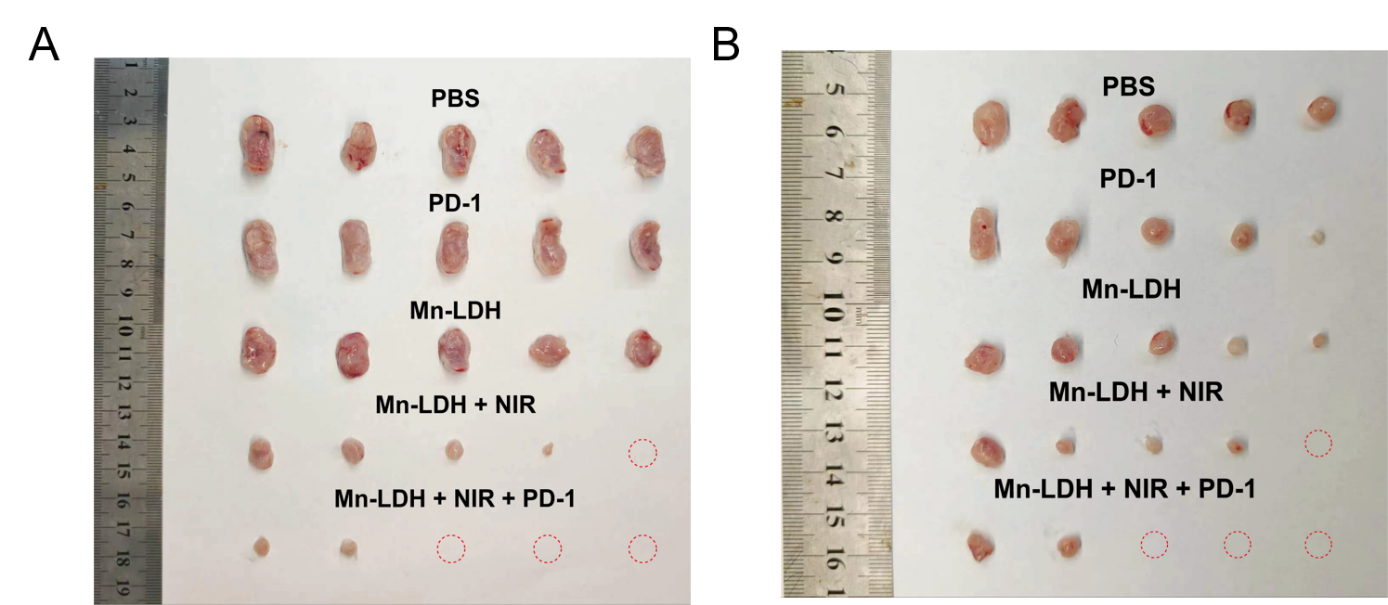


Figure S30. Photograph of excised primary (A) and secondary (B) 4T1 tumors (n = 5).
